# Natural Borneol, a Monoterpenoid Compound, Potentiates Selenocystine-Induced Apoptosis in Human Hepatocellular Carcinoma Cells by Enhancement of Cellular Uptake and Activation of ROS-Mediated DNA Damage

**DOI:** 10.1371/journal.pone.0063502

**Published:** 2013-05-20

**Authors:** Jianyu Su, Haoqiang Lai, Jianping Chen, Lin Li, Yum-Shing Wong, Tianfeng Chen, Xiaoling Li

**Affiliations:** 1 College of Light Industry and Food Sciences, South China University of Technology, Guangzhou, China; 2 Department of Chemistry, Jinan University, Guangzhou, China; 3 School of Life Sciences, The Chinese University of Hong Kong, Hong Kong SAR, China; University of Pittsburgh School of Medicine, United States of America

## Abstract

Selenocystine (SeC) has been identified as a novel compound with broad-spectrum anticancer activities. Natural borneol (NB) is a monoterpenoid compound that has been used as a promoter of drug absorption. In the present study, we demonstrated that NB significantly enhanced the cellular uptake of SeC and potentiated its antiproliferative activity on HepG2 cells by induction of apoptosis. NB effectively synergized with SeC to reduce cancer cell growth through the triggering apoptotic cell death. Further mechanistic studies by Western blotting showed that treatment of the cells with NB and SeC activated the intrinsic apoptotic pathway by regulation of pro-survival and pro-apoptotic Bcl-2 family proteins. Treatment of the cells with NB and SeC induced the activation of p38MAPK and inactivation of Akt and ERK. NB also potentiated SeC to trigger intracellular ROS generation and DNA strand breaks as examined by Comet assay. Moreover, the thiol-reducing antioxidants effectively blocked the occurrence of cell apoptosis, which confirmed the important role of ROS in cell apoptosis. Taken together, these results reveal that NB strongly potentiates SeC-induced apoptosis in cancer cells by enhancement of cellular uptake and activation of ROS-mediated DNA damage. NB could be further developed as a chemosensitizer of SeC in treatment of human cancers.

## Introduction

Hepatocellular carcinoma (HCC), the fifth most common cancer in the world and the third most common cause of cancer-related death, are inherently chemotherapy-resistant tumors with the overexpression of multidrug resistance (MDR) genes [1]. Up to now, there is still lack of effective systemic therapies for this disease. The high expression of transporters of MDR proteins in HCC results in enhanced efflux of cellular drugs, poor drug delivery and dose limitation, which constitute the most challenging problems toward the treatment of HCC in clinical oncology [2]. Therefore, in this respect, the development of new effective agents that are capable to selectively killing HCC cells by overcoming MDR constitute an urgent priority. Nowadays, the combination chemotherapy has been found to be an effective strategy that offers possibility to lower the dose of chemotherapeutic drugs and to decrease the side effects. Till now, many natural and synthetic agents, such as selenocompounds, cytotoxic agents [3] or natural medicines [4], have been employed in the synergized treatments of cancers.

Selenium (Se) is an essential trace element of fundamental important to humans and animals. The adequate supplementation of Se can prevent many types of cancers [5]. Accumulating data has been revealed that selenocompounds possessed potent anticancer activities [6], [7]. Studies have also implied that Se may be clinically applied in chemotherapeutic strategies [8], [9]. For instance, experimental studies from Katzenellenbogen et al [10] have showed that Se displayed anti-liver cancer, and could prevent or reduce chemical hepatocarcinogenesis in animals. Moreover, different from other antioxidant nutrients, dietary Se showed a significant beneficial effect by reducing the risk of hepatocellular carcinoma for 50% [11]. However, Se displayed a narrow margin between the beneficial and toxic effects. As an anticancer agent, the effective dose of Se is close to the toxic range, which greatly limits its clinical application. Although regarded as an essential trace element, Se is toxic if taken in excess. Many studies have revealed that the beneficial and toxic effects of Se on human health were strongly dependent on its concentration and chemical forms [5]–[11]. Organic Se species, especially seleno-amino acids, are less toxic and more effective in anticancer than inorganic Se species [5]–[11].

Although Se exerts a significant role in cancer treatment, the exact molecular mechanisms remain elusive. Selenocystine (SeC), a diselenide oxidation product of selenocysteine, was found be able to reduce tobacco-derived nitrosamine-induced lung tumor in A/J mice [7]. In our previous works, we demonstrated that SeC possessed potent anticancer activities with great selectivity between human cancer cells and normal cells [12], and thus displayed application potential in cancer treatment. Moreover, it was found that SeC triggered cancer cell apoptosis by overproduction of intracellular ROS [12], which subsequently led to activation of DNA damage-mediated p53 phosphorylation and inactivation of ERK and Akt signaling. SeC also demonstrated potent *in vivo* anticancer activities through induction of apoptosis [13], [14]. However, the poor stability and low solubility of SeC hindered its cell membrane permeabilization and the further development as an anticancer drug. Therefore, the strategy to enhance the cellular uptake of SeC could be a highly efficient way to achieve anticancer synergism.

Borneol is a monoterpenoid component present in the essential oils of numerous medicinal plants, such as valerian, chamomile and lavender [15]. It has been widely used in food and drug industries, typically in folk medicine in China and India [16]. Generally, there are two types of borneol: synthetic borneol (SB) and natural borneol (NB). SB is racemate, including (+)-borneol and (-)-isoborneol, while NB only contains (+)-borneol. Previous study has been reported that isoborneol exhibited higher mucosa stimulus and hepatotoxicity than (+)-borneol [17]. Therefore, NB could be a safer form of borneol for commercial applications. Interestingly, studies have proved that NB could improve the oral bioavailability of some poorly permeable drugs by modulating the permeability of intestinal mucous or blood-brain barrier [18]. Several mechanisms have been postulated to explain this potency, including increased cell membrane fluidity, disruption of tight junctions, reduction of mucus viscosity and elasticity, inhibition of efflux transporters and solubilization of the drugs [18], [19]. Therefore, in the present study, we aimed to display the ability of NB to synergize with SeC to induce cancer cell apoptosis, and to elucidate the underlying molecular mechanisms accounting for the synergistic effects. Taken together, our results showed that NB strongly potentiated SeC-induced apoptosis in human hepatocellular carcinoma cells by enhancement of cellular uptake, activation of ROS-mediated DNA damage and inactivation of Akt and ERK. This study suggests that NB could be further developed as a chemosensitizer of SeC in treatment of human cancers.

## Materials and Methods

### 1. Materials

Selenocystine was purchased from Sigma-Aldrich (Sigma-Aldrich, St. Louis, MO) and natural borneol (NB) was obtained from the Natural Institute for the Control of Pharmaceuticals and Biological Products, Beijing, China. Reagent kit for single cell gel electrophoresis assay (Comet Assay) was purchased from Trevigen (Gaithersburg, Md). All antibodies used in this study were purchased from Cell Signaling Technology (Beverly, MA).

### 2. Cell Culture, Determination of Cell Viability and Se Cellular Uptake

Hepatocellular carcinoma HepG2 cell line was obtained from American Type Culture Collection (ATCC, Manassas, VA) and maintained in DMEM medium supplemented with fetal bovine serum (10%), penicillin (100 units/ml) and streptomycin (50 units/ml) at 37°C in a humidified (5% CO_2_, 95% air) atmosphere. The cells were seeded in culture plates for 24 h, and then pre-treated with NB for 12 h and co-incubated with SeC for another 24 h to examine the synergistic effects of NB/SeC on HepG2 cells. Cell viability was determined by MTT assay as previously described [20]. Se cellular uptake was determined by ICP-AES method [13].

### 3. Flow Cytometric Analysis

The cell cycle distribution was analyzed by flow cytometric analysis as previously described [20]. Apoptotic cells with hypodiploid DNA contents were measured by quantifying the sub-G1 peak. For each experiment, 10000 events per sample were recorded.

### 4. Tunnel and DAPI Staining

Cells cultured in chamber slides were fixed with 3.7% formaldehyde for 10 min and permeabilized with 0.1% Triton X-100 in PBS. After then, the cells were incubated with 100 µl/well TUNEL reaction mixture containing nucleotide mixture and terminal deoxynucleotidyl transferase (TdT) for 1 hour and 1 µg/ml of DAPI for 15 min at 37°C, respectively. The cells were then washed with PBS and examined under a ﬂuorescence microscope (Nikon Eclipse 80i).

### 5. Single Cell Gel Electrophoresis (Comet Assay)

Single-cell gelelectrophoresis for detection of DNA damage was performed using the Comet assay reagent kit as previously described [13].

### 6. Caspase Activity Assay

Total cell lysates (100 µg/well) were placed in 96-well plates and then specific caspase substrates (Ac-DEVD-AMC for caspase-3, Ac-IETD-AMC for caspase-8 and Ac-LEHD-AMC for caspase-9) were added. Plates were incubated at 37°C for 2 h in darkness and the caspase activity was determined by fluorescence intensity with the excitation and emission wavelengths set at 380 and 440 nm respectively.

### 7. Measurement of ROS generation

The intracellular ROS generation was evaluated by DCF fluorescence assay [21] **.** Relative DCF fluorescence intensity of treated cells was expressed as percentage of control (as 100%).

### 8. Western Blot Analysis

Total proteins of treated cells were harvested by incubating the cells in the lysis buffer obtained from Cell Signaling Technology. The protein concentrations were determined by BCA kit (Sigma-Aldrich) according to the manufacturer’s protocols. SDS-PAGE was performed in 10% tricine gels with equal amounts of protein loaded per lane. After electrophoresis, proteins were transferred from the gel to a nitrocellulose membrane at 110 V for 1 hour, and then the membrane was blocked with 5% nonfat milk in TBST buffer for 1 hour. The membranes were then incubated with primary antibodies at 1∶1000 dilution in 5% nonfat milk over night at 4°C, followed by secondary antibodies conjugated with horseradish peroxidase at 1∶2000 dilution for 1 hour at room temperature. Protein bands were visualized on X-ray film using an enhanced chemiluminescence system (Kodak). *β*-Actin was used to confirm the equal loading and transfer of proteins.

### 9. Statistical Analysis

Experiments were carried out at least in triplicate and repeated three times. All data were expressed as mean ±S.D. Statistical analysis was performed using SPSS statistical package (SPSS 13.0 for Windows; SPSS, Inc. Chicago, IL). The difference between two groups was analyzed by two-tailed Student’s t-test. The difference between three or more groups was analyzed by one-way analysis of variance multiple comparisons. Differences with *P*<0.05 (*) or *P*<0.01 (**) was considered statistically significant. Bars with different characters are statistically different at *P*<0.05 level.

## Results

### 1. NB Enhances Cellular Uptake and *In Vitro* Anticancer Activity of SeC

**Figure 1:**
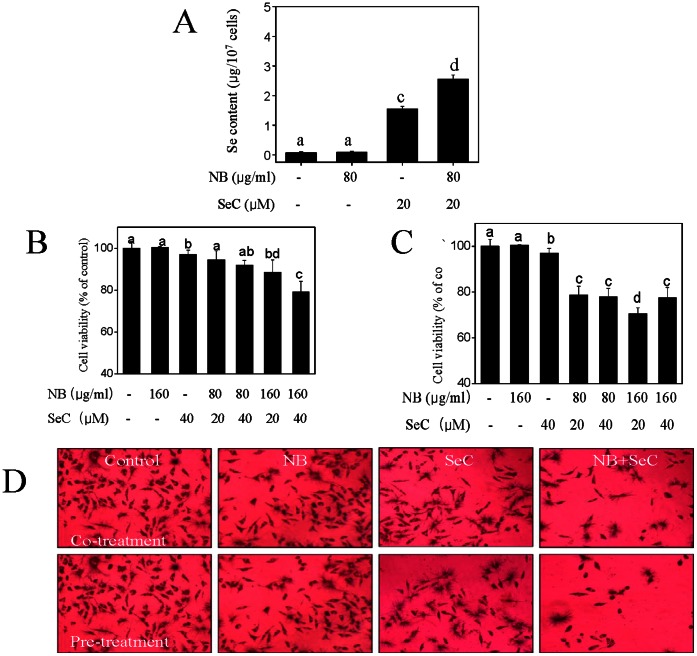
NB enhances the cell growth inhibitory effects of SeC on HepG2 cells. (A) Quantitative analysis of cellular uptake of Se into cells exposed to 80 µg/ml NB and/or 20 µM SeC for 24 h by ICP-AES analysis. (B) Cells growth inhibition induced by the co-treatment of NB and SeC for 24 h and (C) pretreatment of NB for 12 h then incubated with SeC for 24 h. (D) MTT staining image of cells after treatments as examined by light microscopy (magnification 200×).

Firstly, we used the ICP-AES method to investigate the uptake of SeC in HepG2 cells. As shown in **Fig. 1A**, treatment of the cells with NB (80 µg/mL) alone for 36 h showed no effects on the intracellular Se concentration. In contrast, after treatment with 20 µM SeC alone for 24 h, a significant increase in intracellular Se concentration was observed. Interestingly, concomitant treatment with NB and SeC resulted in profound increase in Se concentration in HepG2 cells from 0.07 µg/10^7^ cells (control) to 2.56 µg/10^7^ cells, which was 1.6 fold higher than that of SeC alone. To evaluate the effects of Se uptake on the cell growth status of HepG2 cells, the overall cell growth was determined by employing MTT assay. As illustrated by **Figure.1B and C**, NB alone, even at the concentration of 160 µg/mL, induced negligible growth inhibition on HepG2 cells, while SeC alone only demonstrated a slight inhibition on cancer cell growth. In contrast, co-treatment or pretreatment of the cells with NB (80 µg/mL and 160 µg/mL) significantly enhanced the cells growth inhibitory effects of SeC (20 µM and 40 µM) on HepG2 cells. Interestingly, we found that, pretreatment with NB for 12 h demonstrated higher anticancer activity than those of co-treatment, and pretreatment for 3, 6 and 24 h (data not shown). The results of microscopic examination of cells after MTT staining (**Figure.1D**) also indicated the significant cell growth inhibition after treatments with NB and SeC in combination. What’s more, the effects of pretreatment was much higher then the co-treatment or the individual drug alone. Taken together, these results suggest that NB enhances cellular uptake and *in vitro* anticancer activity of SeC.

### 2. NB Enhances SeC-induced Apoptosis with the Involvement of Caspase Activation

**Figure 2:**
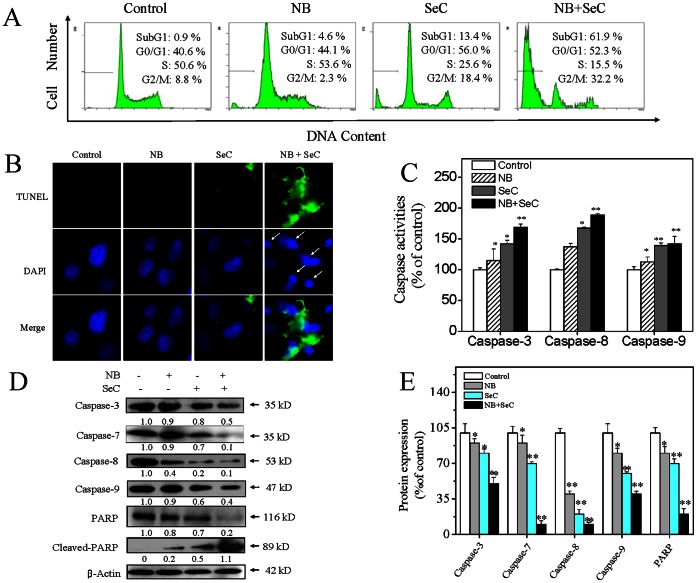
NB enhances the apoptosis-inducing effects of SeC. (A) NB enhances SeC induces apoptosis in HepG2 cells. Cells after treatment were harvested and fixed with 70% ethanol before being stained with propidium iodide. Apoptotic cells with hypodiploid DNA content were measured by quantifying the Sub-G1 peak. (B) Representative photomicrographs of DNA fragmentation and nuclear condensation in response to the co-treatment, as detected by TUNEL assay and DAPI staining. Cells were pre-treated with 80 µg/ml NB for 12 hours followed by treated with 20 µM SeC for another 24 hours (Magnification, 200×). All data here are expressed as means ± SD of triplicates. All images shown here are representative of three independent experiments with similar results. Bars with different characters (a, b, c and d) are statistically different at *P*<0.05 level. (C) Caspase activities as measured by specific fluorescent substrates for caspase-3/8/9. Significant difference between treatment and control groups is indicated at *P*<0.05 (_*_) and *P*<0.01 (_**_) levels. (D) Western blot analysis of caspases activation and PARP cleavage in HepG2 cells treated with NB and SeC for 24 h. Equal loading was confirmed by stripping immunoblots and reprobing for β-actin. All result shown here are representative of three independent experiments with similar results. (E) Protein expression of caspases and PARP as percentage of control. Significant difference between treatment and control groups is indicated at *P*<0.05 (_*_) and *P*<0.01 (_**_) levels.

Inhibition of proliferation in cancer cells could be the result of induction of apoptosis or cell cycle arrest or a combination of these two modes. In order to confirm the action mechanisms of cell death induced by SeC/NB, we carried out a PI-flow cytometric analysis to examine the apoptotic Sub-G1 fraction in the treated cells after treatments. As shown in **Figure 2A**, NB (80 µg/mL) and SeC (20 µM) alone slightly increase the cell apoptosis from 0.9% to 4.6% and 13.4%, respectively. However, the combined treatment with SeC (20 µM) and NB (80 µg/mL) dramatically caused 61.9% of cell apoptosis. To further confirm the induction of apoptosis, we detected the DNA fragmentation and nuclear condensation as apoptotic markers by TUNEL and DAPI co-staining assay. As shown in **Figure 2B**, the cells treated with NB and SeC in combination showed significant DNA fragmentation and nuclear condensation, which were not detected in the cells treated with NB or SeC alone. These results indicate that apoptosis is the major mode of cell death induced by combined treatment of NB and SeC.

Since NB could amplify the *in vitro* anticancer and apoptosis-inducing effects of SeC, we next conducted further experiments to understand the molecular mechanism by which NB sensitizes the cancer cells to SeC. Caspases, a family of cysteine acid proteases, are known to act as important mediators of apoptosis and contribute to the overall apoptotic morphology by cleavage of various cellular substrates. In this study, activation of two initiator caspases, caspase-8 (Fas/TNF-mediated) caspase-9 (mitochondrial-mediated), and an executor caspase caspase-3 were therefore measured by fluorometric assay. **Figure 2C** showed that NB and SeC alone slightly increased the activation of caspase-3/8/9, indicating that both extrinsic death receptor-mediated and intrinsic mitochondria-mediated apoptotic pathways were involved in cell apoptosis induced by either NB or SeC. Furthermore, the combined treatment of NB and SeC synergistically enhanced the activation of caspase-3/8/9. Thess results were further confirmed by cleavage of caspases and PARP as examined by Western blotting. As shown in **Figure.2D and 2E**, exposure of HepG2 cells to combined treatment of NB and SeC resulted in cleavage of caspase-3/7, which subsequently induced the proteolytic cleavage of PARP, a protein serving as a biochemical hallmark of cells undergoing apoptosis. Taken together, these results demonstrated that both the extrinsic and intrinsic apoptosis pathways were involved in the combined treatment-induced apoptosis in HepG2 cells.

### 3. NB and SeC Trigger Mitochondria Dysfunction by Regulating the Expression of Bcl-2 Family Proteins

**Figure 3:**
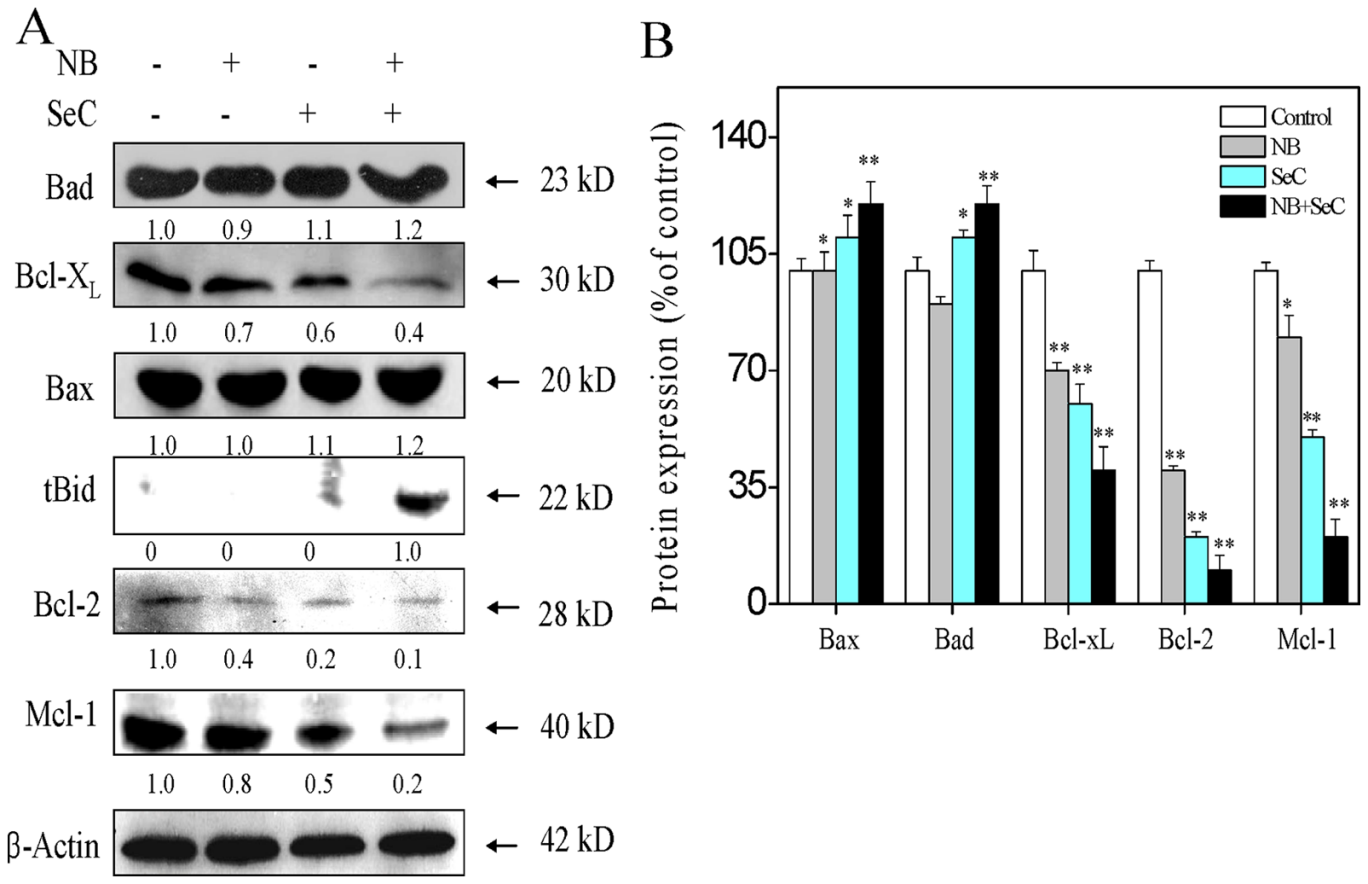
Effects of NB in combination with SeC on the expression level of Bcl-2 family proteins. (A) Western blot analysis of Bcl-2 family protein. Cells were pretreated with 80 µg/ml NB for 12 h and then incubated with 20 µM SeC for another 24 h. (B) Protein expression level of Bcl-2 family as percentage of control. All result shown here are representative of three independent experiments with similar results. Significant difference between treatment and control groups is indicated at *P*<0.05 (_*_) and *P*<0.01 (_**_) levels.

Mitochondria play a central role in regulation of cell fate by integrating the apoptotic signals originated from both the intrinsic and extrinsic apoptosis pathways. The depletion of mitochondrial membrane potential (ΔΨm) is a crucial step in the apoptotic process and is lethal to the cells, because it leads to the release of diverse apoptogenic factors from mitochondria into cytoplasm. Bcl-2 family proteins have been described as critical regulators of the mitochondrial apoptosis pathway. Therefore, we examined the effects of NB and SeC on the expression levels of pro-survival and pro-apoptotic Bcl-2 family proteins in HepG2 cells by Western blotting. As shown in **Figure 3**, treatments of the cells with NB and SeC increased the total expression levels of the pro-apoptotic protein Bad and Bax, but decreased the expression level of the anti-apoptotic protein Bcl-X_L_, Bcl-2 and Mcl-1. Truncation of Bid (tBid), a death agonist member of BH3 domain-only protein family, is an activated form of Bid that possesses potent pro-apoptotic activity. Upon activation, tBid could translocate from the cytoplasm to mitochondria membrane and induce oligomerization of Bak and Bax, thereby promoting the release of mitochondrial proteins into cytoplasm [21]. By using Western blotting, we showed that, the combined treatment of cells with NB and SeC induced a significant increase in truncated Bid, which confirmed the activation of the extrinsic apoptosis pathway. This is consistent with the activation of caspase-8 in response to apoptotic signal (**Figure. 2B**). Taken together, these results demonstrate that the enhancement of SeC-induced apoptosis by NB is primarily correlated with down-regulation of Bcl-2/Bax and Bcl-xL/Bad expression ratio and the truncation of Bid.

### 4. NB and SeC Synergize to Induce DNA Damage-mediated p53 Phosphorylation

**Figure 4:**
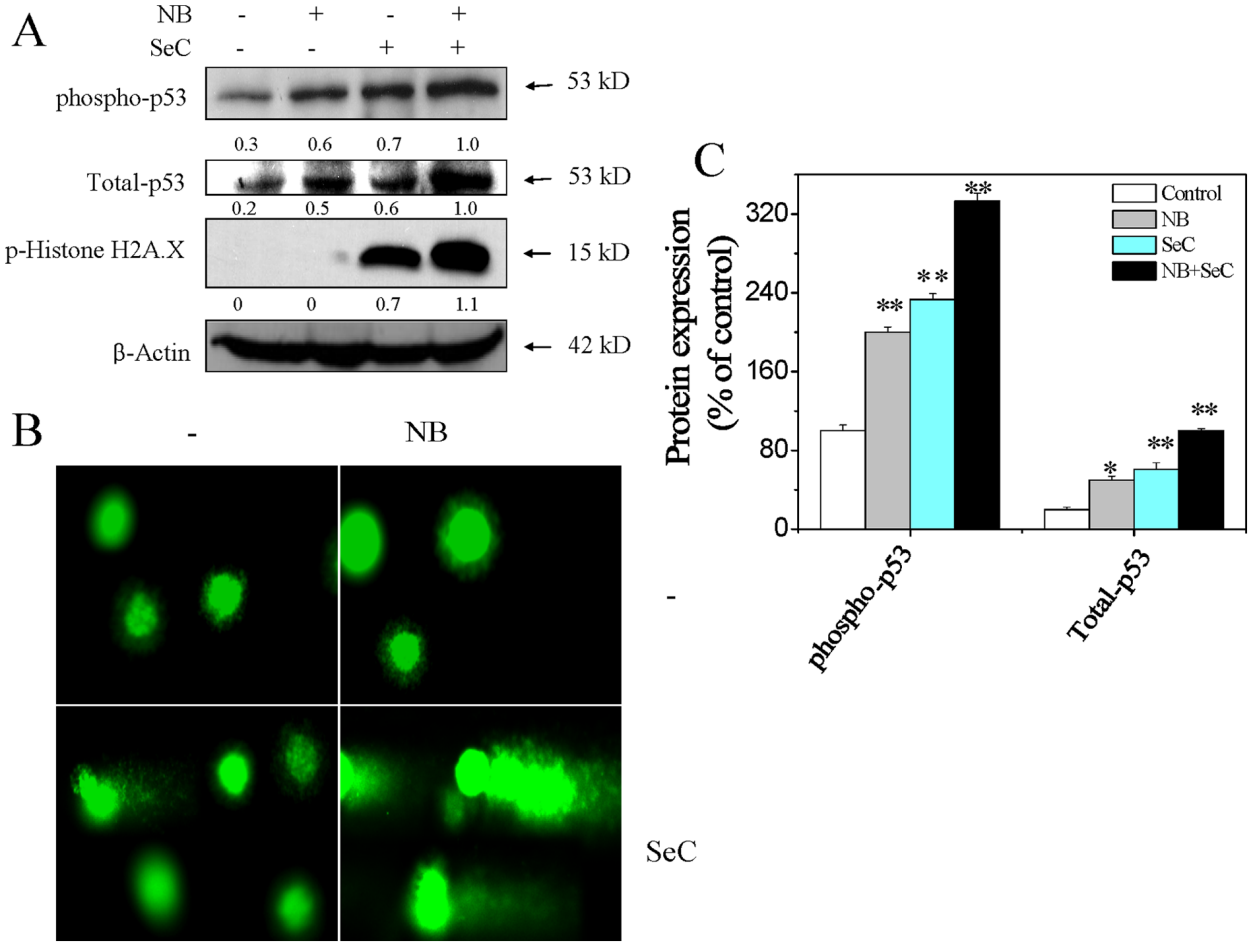
DNA damage-mediated p53 activation induced by NB and SeC. (A) NB enhances SeC-induced up-regulation of phosphorylated p53, total p53 and histone. Cells were pretreated with 80 µg/ml NB for 12 hours and incubated with 20 µM SeC for another 24 hours. The expression levels of the tested proteins were examined via Western blotting method. (B) NB enhances SeC-induced DNA damage as evidenced by Comet assay at the single-cell level. Cells after treatment were immediately analyzed by Comet assay as described in section of Materials and methods. The length of tail reflects the DNA damages in cells. All images shown here are representative of three independent experiments with similar results. (C) Protein expression level of phospho-p53 and Total-p53 as percentage of control. Significant difference between treatment and control groups is indicated at *P*<0.05 (_*_) and *P*<0.01 (_**_) levels.

Many studies have showed that selenocompounds exhibited the potential to induce DNA damage, and thus activated p53 signaling pathway [13]. P53 is involved in both the extrinsic and intrinsic apoptosis pathways with the involvement of mitochondrial depolarization. In our previous studies, we have showed that SeC could induce DNA damage by triggering the overproduction of intracellular ROS [13]. Moreover, SeC up-regulated the expression of total and phosphorylated p53 in cancer cells [22]. Therefore, we investigated the potentiation of SeC-induced DNA damage by NB at single cell level by employing Comet assay. Firstly, the results of immunoblotting assay revealed that NB and SeC in combination markedly increased the expression levels of total and phosphorylated p53 at Ser15 site, and p-Histone H2A.X at Ser 139 site, which indicated the synergistic amplification of DNA damage by NB on SeC (**Figure 4A and 4C**). This finding was further confirmed by Comet assay, which is based on the ability of denatured or cleaved DNA fragments to migrate out of the cell under the inﬂuence of an electric field. As shown in **Figure 4B**, after treatment for indicated time, NB did not induce apoptotic DNA fragmentation for lacking tail DNA, while only slight DNA damage was observed in cells exposed to SeC alone. Interestingly, combination of NB and SeC resulted in much higher level of DNA fragmentation, as evidenced by the increase of tail DNA. Collectively, these results support that, enhancement of DNA damage contributes to the synergistic effects of NB and SeC on HepG2 cells.

### 5. Involvement of Akt and MAPKs Pathways in the Synergistic Effect of NB and SeC

**Figure 5:**
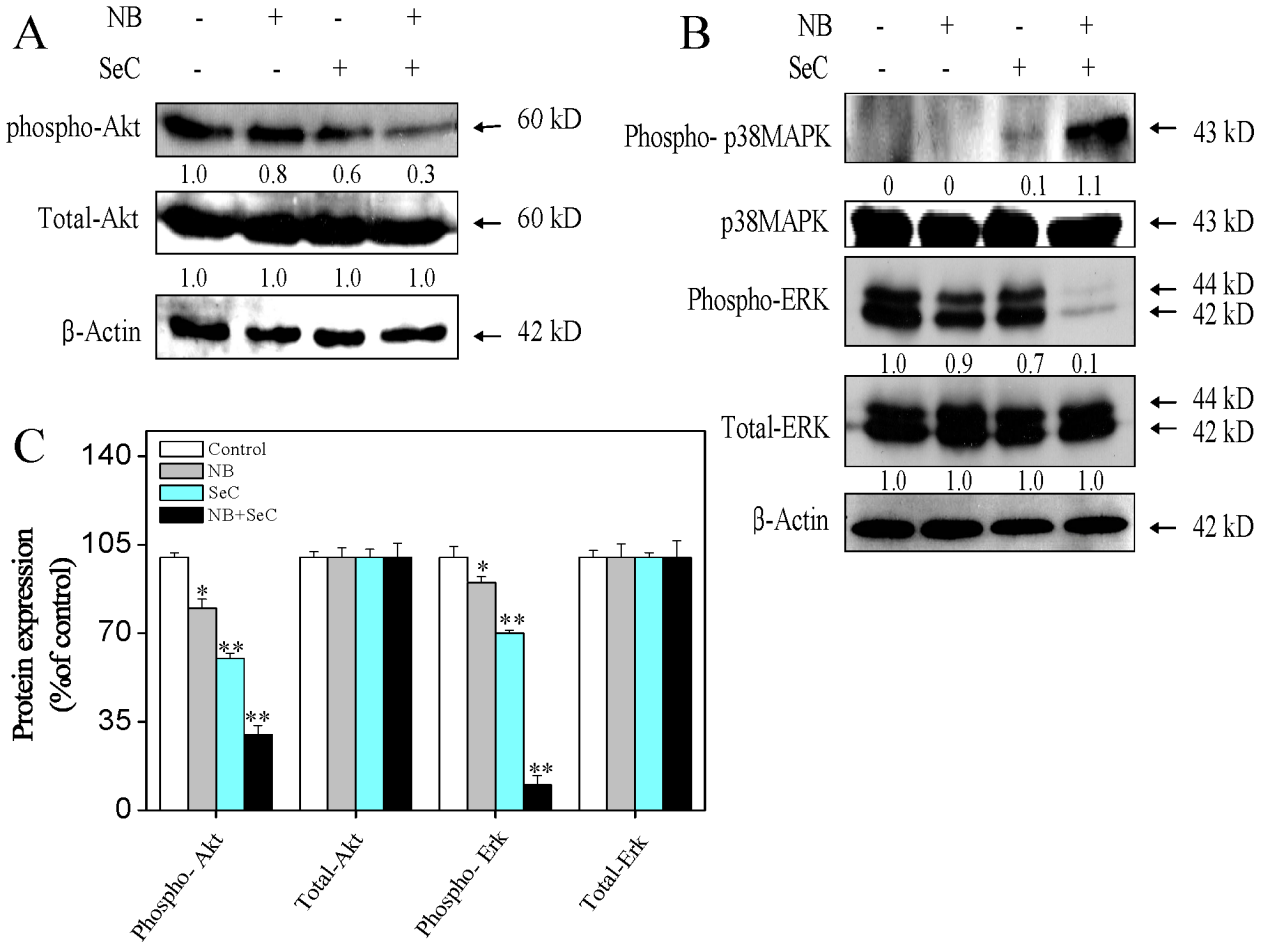
Effects of NB and SeC on Akt and MAPK signaling pathways. (A) Effects of NB and SeC on the phosphorylation status and the expression level of Akt. Cells were treated with indicated concentrations of NB and SeC for corresponding time. (B) The phosphorylation status and expression levels of p38 MAPK and ERK after treatment of NB and SeC. The immunoblots were representatives of three independent experiments with similar results. (C) Expression of MAPKs family and Akt proteins as percentage of control. Significant difference between treatment and control groups is indicated at *P*<0.05 (_*_) and *P*<0.01 (_**_) levels.

MAPk signaling pathway plays an important role in regulating cell cycle progression and proliferation through transmitting extracellular signals from the cell membrane to the nucleus. The serine/threonine kinase Akt/PKB is implicated in the maintainance of cell survival by blocking the function of pro-apoptotic proteins. Akt/PKB could promote cell growth through multiple downstream targets implicated in cell-cycle regulation [23]. In our previous studies, we have showed that SeC activated MAPKs and inactivated the phosphorylation of Akt and ERK1/2 in MCF-7 cells [14]. Therefore, we investigated the contribution of Akt and MAPKs signal pathways to the cell apoptosis induced by NB and SeC by immunoblotting assay. As shown in **Figure.5**, comparing with each drug alone, pretreatment of the cells with NB significantly enhanced SeC-induced dephosphorylation of Akt and ERK1/2, with no obvious change in total Akt and ERK1/2 observed. Furthermore, NB exhibited no effect on the expression of phosphorylated p38 MAPK, while only a slight elevation of phosphorylated p38MAPK was observed in cells exposed to SeC alone. Whereas, NB and SeC in combination significantly induced a rapid augment in the expression level of phosphorylated p38 MAPK without any variation on total expression level of phosphorylated p38 MAPK. Taken together, NB potentiated SeC to induce apoptosis with involvement of down-regulation of Akt and ERK1/2 phosphorylation and up-regulation of phosphorylated p38 MAPK.

### 6. NB Enhances SeC-induce Cell Apoptosis via ROS Generation

**Figure 6:**
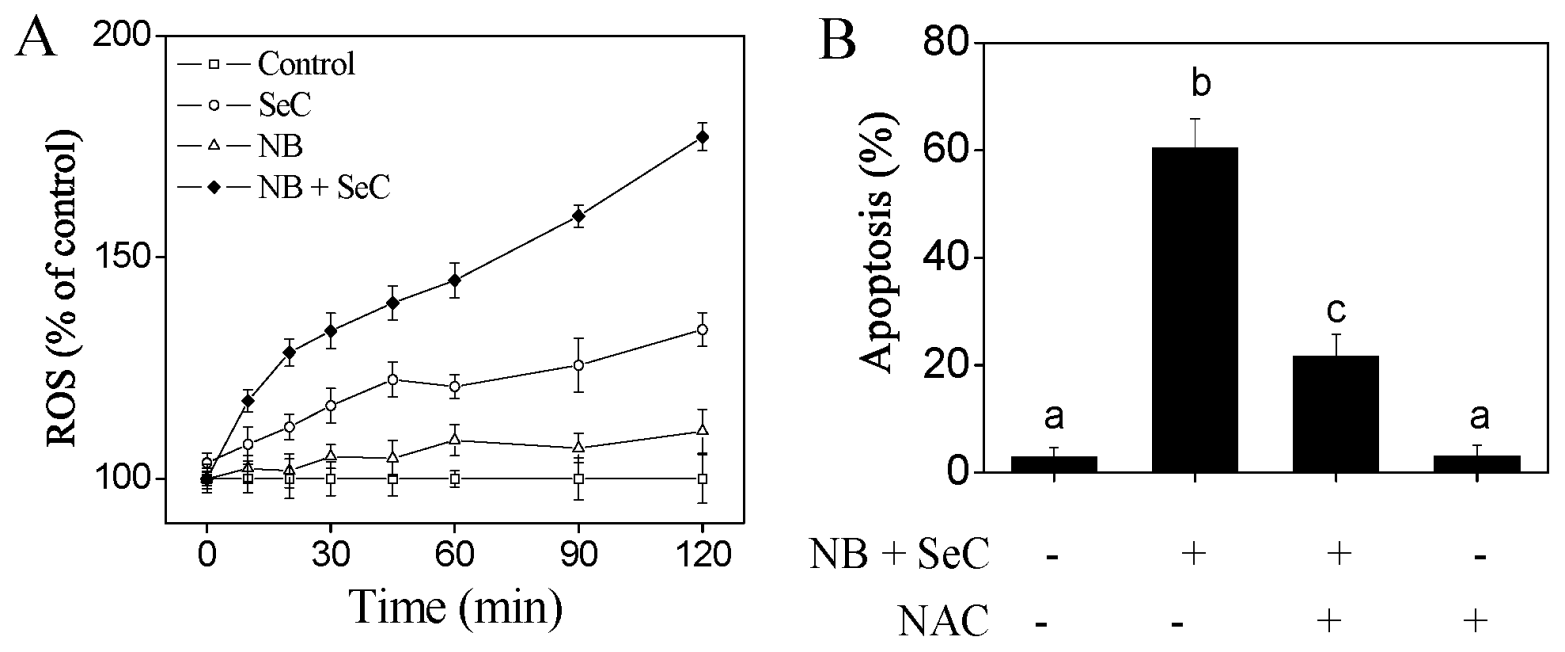
The role of intracellular ROS generation in HepG2 cell apoptosis induced by NB and SeC in combination. (A) Cells were exposed to NB and SeC at indicated concentration for different times and the levels of the intracellular ROS were analyzed by measuring the fluorescence intensity of an oxidation-sensitive fluorescein DCFH-DA. Cells were treated with 80 µg/ml NB with or without 20 µM SeC for different periods of time. (B) Effects of NAC on cell apoptosis induced by NB and SeC. Cells were pretreated with 2 mM NAC or for 4 h prior to addition of NB and SeC. Apoptotic cell death was determined by flow cytometric analysis. All results were obtained from three independent experiments. Bars with different characters (a, b, and c) are statistically different at the P<0.05 level.

ROS, mainly including hydrogen peroxide, superoxide, and hydroxyl radical, is well known to be produced from normal cellular oxygen metabolism. It has been reported that ROS is involved in cancer cell apoptosis induced by chemotherapeutic agents and radiotherapy [24]. In our previous studies, we have showed that SeC was capable of inducing ROS generation in many cancer cells including HepG2 cells [12]. Therefore, we decided to detect whether NB was able to synergize with SeC to trigger ROS generation by measuring the DCF fluorescence intensity. The results showed that pretreatment of the cells with NB significantly enhanced SeC-induced ROS generation a time-dependent manner (**Figure 6A**). To further confirm the important role of ROS generation in cell apoptosis, we next investigated the effects of a thiol-reducing antioxidant (NAC) on the apoptotic cell death. As revealed in **Figure 6B**, NAC effectively blocked the cells apoptosis induced by NB and SeC in combination, which indicate that ROS is the upstream mediator of apoptosis. Taken together, these results suggest that NB synergizes with SeC to induce cancer cell apoptosis in a ROS-dependent manner.

## Discussion

HCC has been considered as a chemotherapy-resistant tumor due to the overexpression of MDR genes, which lead to the failure of chemotherapeutic drugs to induce cancer cell apoptosis. Therefore, design and discovery of new effective apoptosis-inducing agents without toxic effects have kindled great interest of scientists. Nowadays, synergistic drug treatment has been recognized as an effective strategy to achieve favorable outcome for cancer chemotherapy and chemoprevention. In this study, we demonstrated that NB could synergize with SeC to induce apoptosis in cancer cells.

Apoptosis plays an important role in the development, homeostasis, and prevention of cancer. Nowadays, induction of apoptosis has been regarded as the major cytotoxic mechanism of cancer chemotherapy and chemoprevention [25]. Upon activation, apoptosis occurs through mitochondria-mediated (intrinsic) pathway and death receptor-mediated (extrinsic) pathway [26]. These two pathways exert their roles by activating various caspase cascades. Under specific situation, the extrinsic pathway takes a crosstalk with the intrinsic pathway through the truncation of Bid, which relayed the apoptotic signal from the cell surface to mitochondria and triggered the self-killing process [21]. Induction of apoptosis has been identified as a critical mechanism accounting for the anticancer action of selenocompounds, especially SeC [13]. SeC could induce apoptotic cell death in various human cancer cells with lower cytotoxicity toward normal cells [12]. In both A375 human melanoma and MCF-7 human breast cancer cells, SeC triggered caspase-mediated and/or caspase-independent apoptosis by activating ROS-mediated mitochondria dysfunction and p53 phosphorylation [13], [22]. However, although potent anticancer effects of SeC have been reported, the poor stability and low solubility of SeC hindered its cell membrane permeabilization and the further development as an anticancer drug. Therefore, the strategy to enhance the cellular uptake of SeC could be a highly efficient way to achieve anticancer synergism.

Generally, solubility and permeability are the fundamental determinants for drug application [27]. Toward poorly absorbable drugs used in clinical trails, different types of absorption enhancer have been utilized, such as surfactants, bile salts, chelating agents, fatty acids and essential oils [28]. NB, a bicyclic monoterpenoid alcohol present in the essential oils of numerous medicinal plants, such as valerian, chamomile and lavender [15], is often used in food and drug industries and has also been reported to be an safe agent for clinical usage [29]. As an absorption promoter, NB could increase the mucosa absorption independently or co-administration with other adjuvants [18]. Interestingly, in the present study, we found that, NB effectively promoted the SeC cellular uptake and enhanced SeC-induced apoptosis in human cancer cells.

Cancer cells resist to chemotherapeutic drugs through overexpression of multidrug resistance proteins. P-glycoprotein (P-gp), one of the most well-characterized drug efflux pumps [30] that was overexpressed in many cancer cell lines including HepG2 cells [31], was able to pump out cytotoxic agents from the cytosolic compartment, thus lowering the intracellular drug concentrations. Inhibition of the P-gp could prevent the P-gp-mediated MDR phenotype and improve the effectiveness of chemotherapy [32]. NB has been found be able to inhibit P-gp activity and enhance the solubility of the drugs [33]. After passing through the gastrointestinal mucous membrane, NB could modulate the epithelial junction permeability and promote the paracellular drug transportation [34]. In the present study, we have confirmed that, NB significantly enhanced the intracellular uptake of SeC in HepG2 cells (**Figure 1A**). Probably, NB may inhibit the P-gp activity and solubilize SeC, as well as enhance SeC transportation by modulating the permeability of cell membrane. The enhanced accumulation of SeC finally triggered apoptosis in HepG2 cells. Caspases activation was crucial for Se-induced apoptosis in many cancer cell lines [35]. In this study, we showed that, NB pretreatment augmented SeC-induce apoptosis through activation of various caspases in HepG2 cells, as evidenced by increase in activities of caspase-3/8/9 and PARP cleavage (**Figure 2**). Moreover, activation of caspase-8 truncated pro-apoptotic Bcl-2 family member Bid (**Figure 3**), which could translocate to mitochondria and activated the intrinsic apoptotic pathway. These results demonstrated that both the extrinsic and intrinsic apoptosis pathways and their crosstalk were involved in the combined treatment-induced apoptosis in HepG2 cells.

Mitochondria have been postulated to be a centre point for converging both extrinsic and intrinsic apoptosis signals. The mitochondrial apoptosis pathway is tightly regulated by Bcl-2 family proteins that comprise both pro-apoptotic proteins, such as Bax, Bad and Bid, and anti-apoptotic proteins, such as Bcl-2 and Bcl-xL. Bcl-2 and Bcl-xL bind to the outer membrane of mitochondria and block cytochrome c efflux [36]. Generally, these pro- and anti-apoptotic proteins are kept in balance, which commitment to stabilize the integrality membrane of mitochondria [37]. Once the disruption of this balance occurs, apoptosis can be initiated through mitochondria-mediate pathway. In many cancer cell types, Bcl-2 and Bcl-X_L_ were overexpressed, which contributed to the resistance of cancer cells to chemotherapeutic agents and radiation therapy [38]. Therefore, new small molecules have been designed to antagonists Bcl-2 and Bcl-X_L_, such as ABT-263 [39]. Results from this study showed that NB synergized with SeC to inhibit the expression of Bcl-2, Bcl-X_L_ and Mcl-1 (**Figure 3**). The combined treatment also up-regulated the expression levels of Bad and Bax. Therefore, the distinct down-regulation of the Bcl-2/Bax and Bcl-X_L_/Bad expression ratio could be a predominant mechanism by which NB in combination with SeC induce mitochondria-mediated apoptosis in HepG2 cells.

ERK and Akt signaling pathways have been confirmed to play important roles in cell proliferation, drugs resistance and cell apoptosis [40]. Both of these two signaling pathways in melanoma are constitutively activated. Akt enhances the survival of cells by blocking the function of pro-apoptotic proteins, such as BAD, FOXO, p53, GSK3 isoforms and caspase-9 [41]. Similar to Akt, ERK could control diverse cellular processes, such as proliferation, survival, differentiation and motility [42]. It could also prevent cell apoptosis by blocking the activation of caspases [43] and enhance the expression of anti-apoptotic factors such as Mcl-1 [44]. Contrary to ERK and Akt, p38 MAPK has been implicated to mediate apoptosis in response to a variety of external stimuli [45]. With regard to the crucial role of Akt and MAPKs signaling pathways in determination of cell fate, many strategies have been postulated for treatment of melanoma by targeting these pathways [40], [46]. Moreover, accumulative evidence supported that selenocompounds exerted their apoptosis-inducing activities in cancer cells by regulating the MAPK and PI3K/Akt pathways [14]. In this study, we showed that NB and SeC obviously up-regulated the phosphorylation of p38MAPK, but decreased the expression levels of phosphorylated Akt and ERK (**Figure 5**). These results elaborate the underlying mechanisms accounting for the synergistic apoptosis-inducing effects of NB and SeC in HepG2 cells.

Recently, ROS has been identified as potential modulators of apoptosis by regulating both of the extrinsic and intrinsic apoptosis pathways [24]. ROS may participate in the activation of death receptor mainly through inducing receptor clustering and formation of lipid-raft-derived signaling platforms [47]. Oxidative stress could stimulate increase in metabolic activity and mitochondrial malfunction, which promotes the release of apoptogenic facotrs from mitochondria inner membrane space and initiates apoptotic cascades. Overproduction of ROS results in accumulation of oxidative products of DNA, such as DNA strand breaks (DSBs), DNA intra-strand adducts and DNA–protein crosslinks [47]. In response to DSBs, ATM and ATR phosphorylate various downstream substrates, such as CHK1 and CHK2, H_2_AX and p53, to trigger cell apoptosis. Activation of p53 surpasses a particular threshold and activates pro-apoptotic genes such as Bax, PUMA, Noxa and Bid [48]. It has been reported that p53 could bind to Bcl-2 and Bcl-X_L_ at the membrane of mitochondria, thereby promoting mitochondrial destabilization [49]. In this study, we showed that, NB pretreatment significantly elevated the phosphorylation of Histone H2A.X and p53, and enhanced the SeC cellular uptake and ROS accumulation. Moreover, significant DSBs inevitably caused the activation of p53 pathway, which directly down-regulated the expression levels of Bcl-2 and Bcl-X_L_ and thus augmented the apoptotic signal (**Figure 4** **–** **6**). Furthermore, it was showed that, thiol-reducing antioxidants effectively blocked the cell apoptosis induced by NB and SeC, which reveals the important role of ROS in the cancer cell apoptosis triggered by NB and SeC.

**Figure 7:**
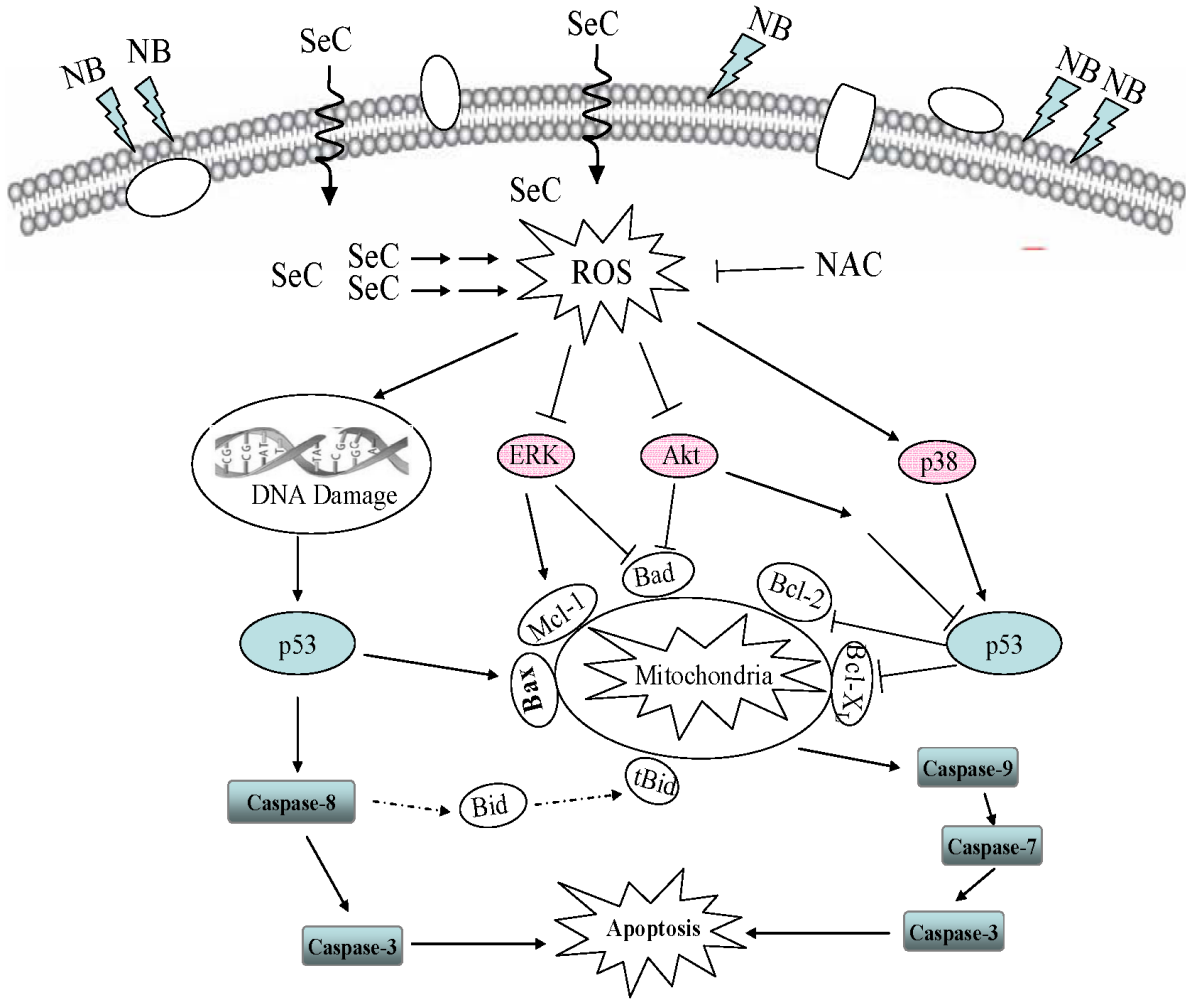
Proposed signaling pathway triggered by NB and SeC in combination in HepG2 cells. NB enhances the cellular uptake of SeC, which subsequently augmented intracellular ROS generation and DSBs. DNA damage activates p53 pathway, which causes mitochondrial dysfunction by regulating the expression levels of Bcl-2 family members. Mitochondrial dysfunction results in release of apoptogenic factors into cytosol, and then activates several caspase cascades, and finally leads to cell apoptosis. In addition, as a functional loop, ROS produced in mitochondria promotes the phosphorylation of p38MAPK and inactivation of Akt and ERK, which enhances the p53 activation and potentiates the apoptotic cascade.

Based on the above results, we proposed a signaling network for the synergistic action of NB and SeC. As depicted in **Figure 7**, NB pretreatment enhances the cellular uptake of SeC, which subsequently augmented intracellular ROS generation and DSBs. DNA damage activates p53 pathway, which causes mitochondrial dysfunction by regulating the expression levels of pro-survival and pro-apoptotic Bcl-2 family members. Mitochondrial dysfunction results in release of apoptogenic factors into cytosol, and then activates several caspase cascades, and finally leads to cell apoptosis. In addition, as a functional loop, ROS produced in mitochondria promotes the phosphorylation of p38MAPK and inactivation of Akt (Eventhough low level ROS play an important role in normal cells proliferation through amplifying Akt/mTOR signal pathway [50], however, once the accumulation of ROS above a certain threshold, apoptosis may be induced through blocking the activation of Akt [51]. In this present study, we found that SeC is capable of inducing ROS generation in a time-dependent manner, which is concomitant with previous study [12], interestingly, pretreatment of NB synergized SeC induce more significant HepG2 cells apoptosis through triggering ROS overproduction, therefore, phosphorylation of Akt may be infected by ROS and potentiates the apoptosis cascade in this synergism.) and ERK, which enhances the p53 activation and potentiates the apoptosis cascade. Taken together, our results showed that NB strongly potentiated SeC-induced apoptosis in human hepatocellular carcinoma cells by enhancement of cellular uptake, activation of ROS-mediated DNA damage and inactivation of Akt and ERK. This study suggests that NB could be further developed as a chemosensitizer of SeC in treatment of human cancers.
