# Expression of Concern: Natural borneol, a monoterpenoid compound, potentiates selenocystine-induced apoptosis in human hepatocellular carcinoma cells by enhancement of cellular uptake and activation of ROS-mediated DNA damage

**DOI:** 10.1371/journal.pone.0336879

**Published:** 2025-12-01

**Authors:** The PLOS One Editors

Following the publication of this article [1], concerns were raised regarding the results presented in Figs 1-3 and 5, and the cell line used. Specifically,

- In Fig 1D, the following panels appear to overlap:
  - ◦ Co-treatment control and pre-treatment control
  - ◦ Co-treatment NB and pre-treatment NB
- The Fig 2D, 3A, and 5B β-actin panels of this article [1] appear similar to the Fig 8C β-actin panel of [2,3] despite being used to represent different experimental conditions in [1] and [2,3].
- The only cell line reported in this study is the hepatocellular carcinoma HepG2 cell line. The HepG2 cell line has been identified as a misclassified cell line, originally thought to be a hepatocellular carcinoma cell line but shown to originate from a hepatoblastoma instead [4] prior to the publication of [1].

The authors stated that the incorrect panels for pre-treatment control and NB were used during the preparation of Fig 1D, and provided triplicate underlying data from the original experiments in S1 File. The correct panels are presented in the updated Fig 1.

Regarding the β-actin panel overlap concerns, the authors stated that the Figs 2D, 3A, and 5B results originate from the same lysate, that the panels in this article [1] are correct, and that an incorrect panel was used during figure preparation for [2, retracted in 3]. They provided raw blots and individual level data underlying most of the panels presented in Figs 2-5 in S2 File and S3 File.

Regarding the cell line concerns, the authors stated that they obtained the cell line from ATCC and provided STR profiling data to confirm the cell line in S4 File. PLOS notes that the STR report states that the profiled samples were received on 07 July 2022 and this report may not be representative of cells used in a study published in 2013. As this study only reports the use of the HepG2 cell line, the relevance of the published results with regards to hepatocellular carcinoma remains unclear.

The *PLOS One* Editors issue this Expression of Concern to inform readers to interpret the results with caution in light of the misclassified cell line concerns, to provide an updated Fig 1, and to relay the available data provided by the corresponding author.

**Fig 1:**
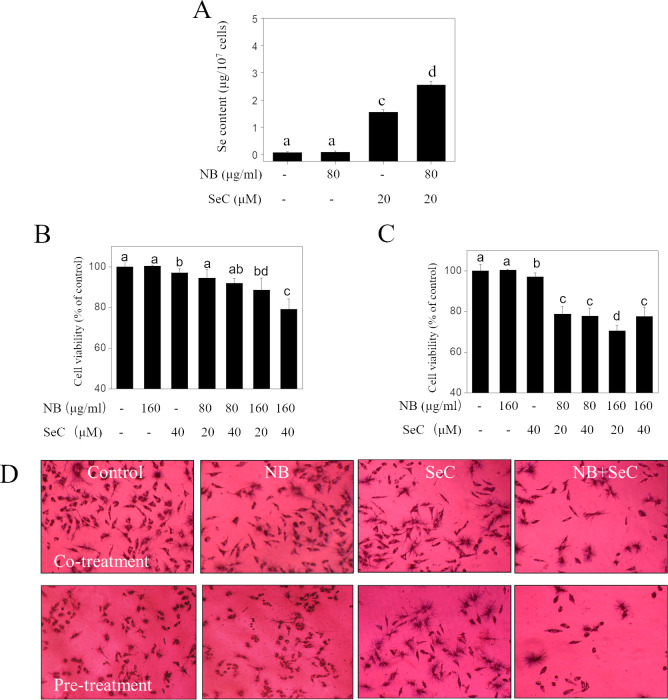
NB enhances the cell growth inhibitory effects of SeC on HepG2 cells. **(A)** Quantitative analysis of cellular uptake of Se into cells exposed to 80 µg/ml NB and/or 20 µM SeC for 24 h by ICP-AES analysis. **(B)** Cells growth inhibition induced by the co-treatment of NB and SeC for 24 h and (C) pretreatment of NB for 12 h then incubated with SeC for 24 **h. (D)** MTT staining image of cells after treatments as examined by light microscopy (magnification 200×).

## Supporting information

S1 File Triplicate image data underlying Figure 1D. (TIF)

S2 File Blot data underlying Figs 2D, 3A, 4A, 5A, and 5B. (PPTX)

S3 File Individual level data underlying Figs 1B and 1C. (ZIP)

S4 File STR profiling results. (ZIP)
