# Supplementary figures and images for: Expression of Concern: Natural borneol, a monoterpenoid compound, potentiates selenocystine-induced apoptosis in human hepatocellular carcinoma cells by enhancement of cellular uptake and activation of ROS-mediated DNA damage
Source: PLoS One. 2025 Dec 1;20(12):e0336879. doi: 10.1371/journal.pone.0336879 (PMC12668515; doi:10.1371/journal.pone.0336879)

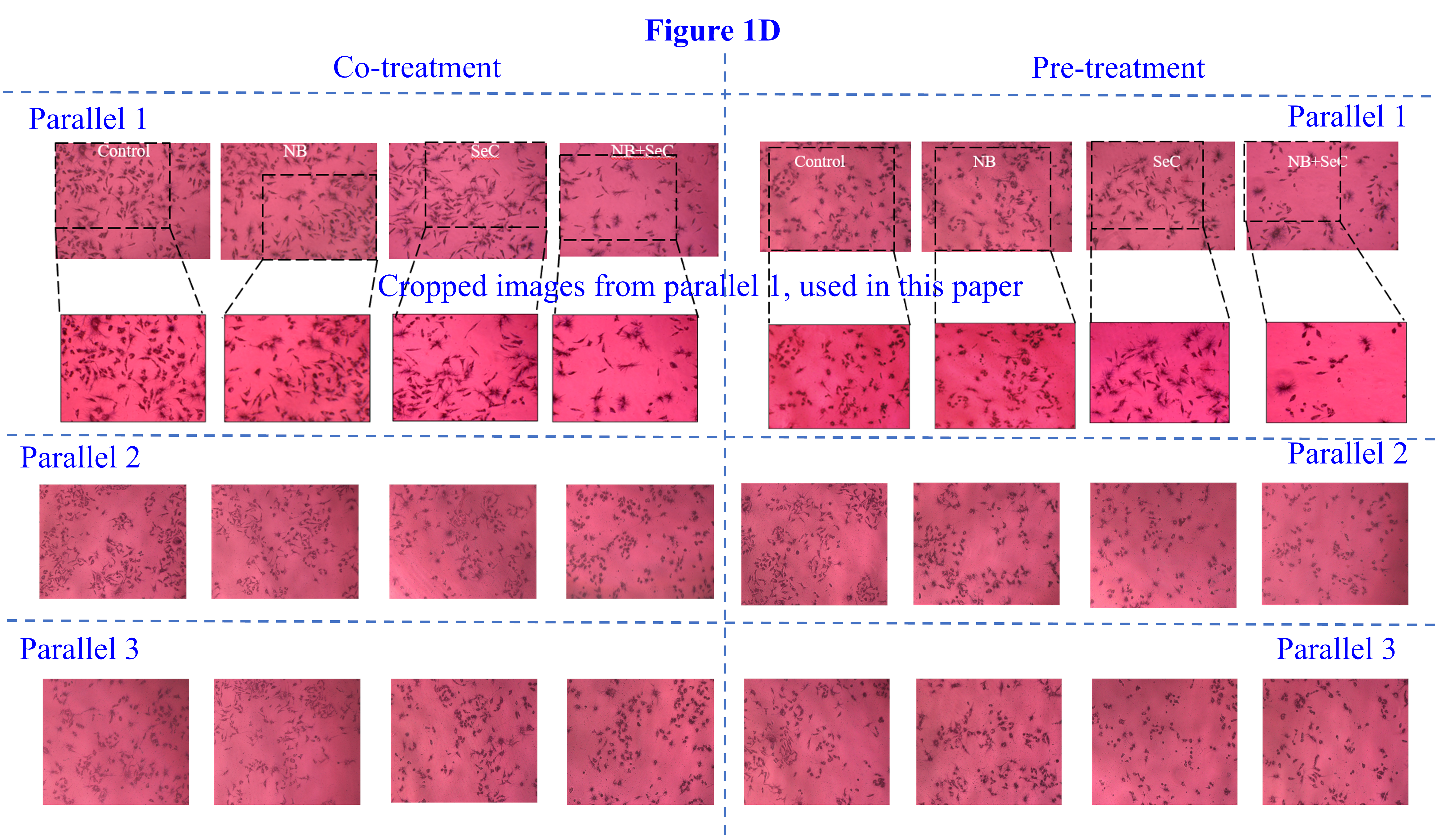

Supplement: S1 File — (TIF) [file pone.0336879.s001.tif]

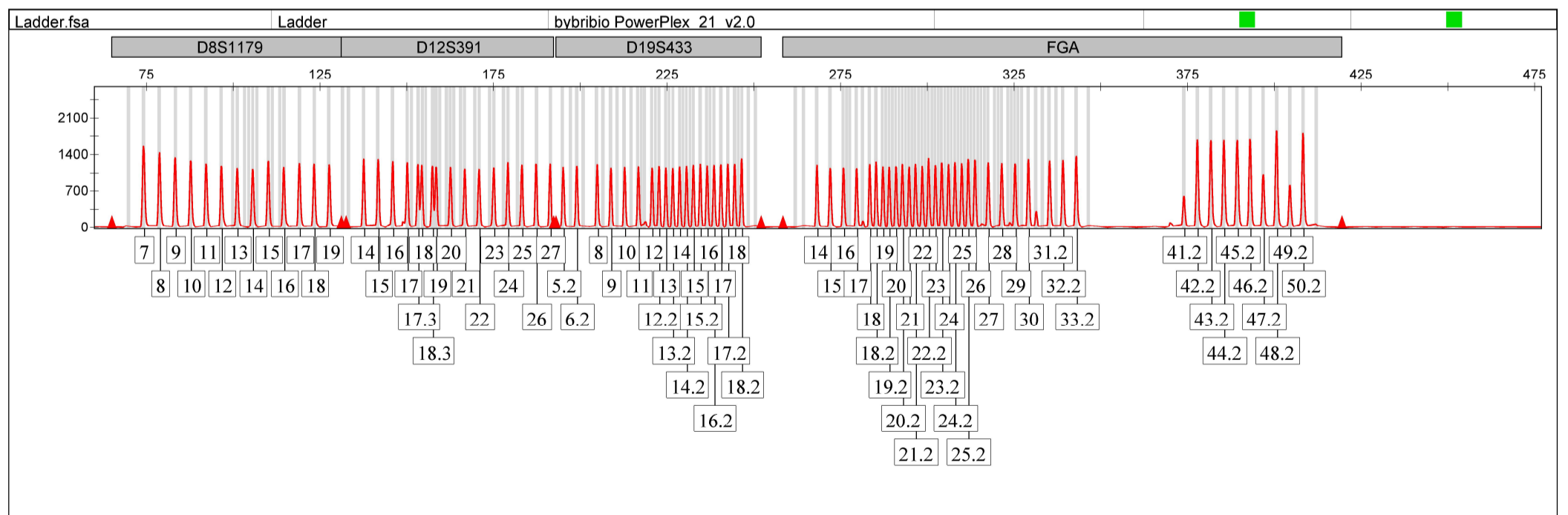

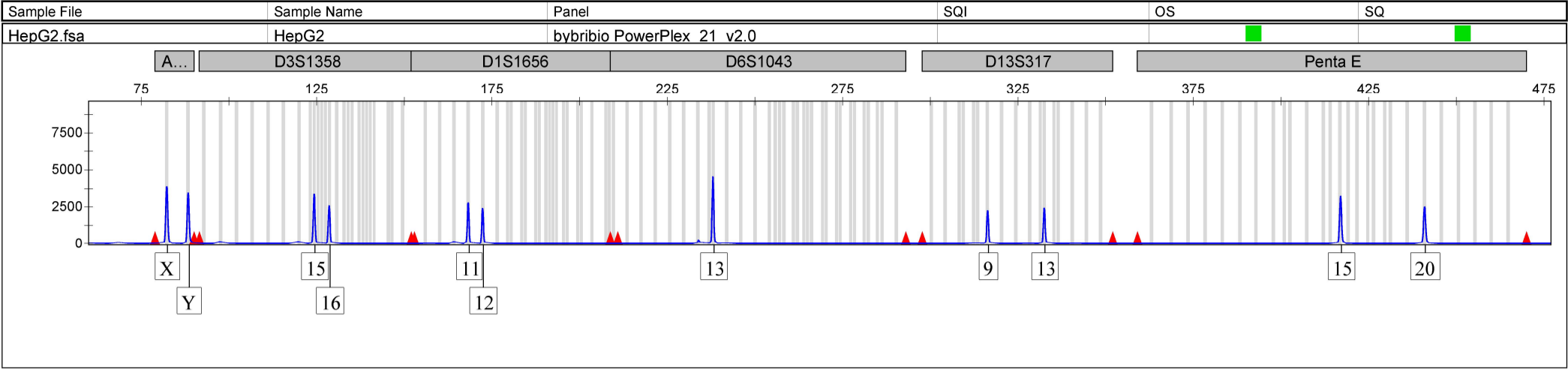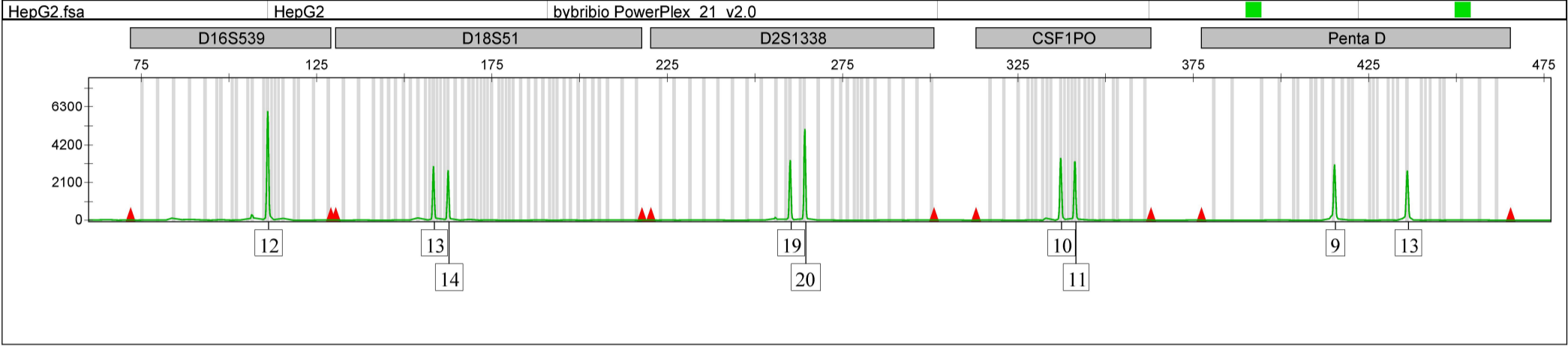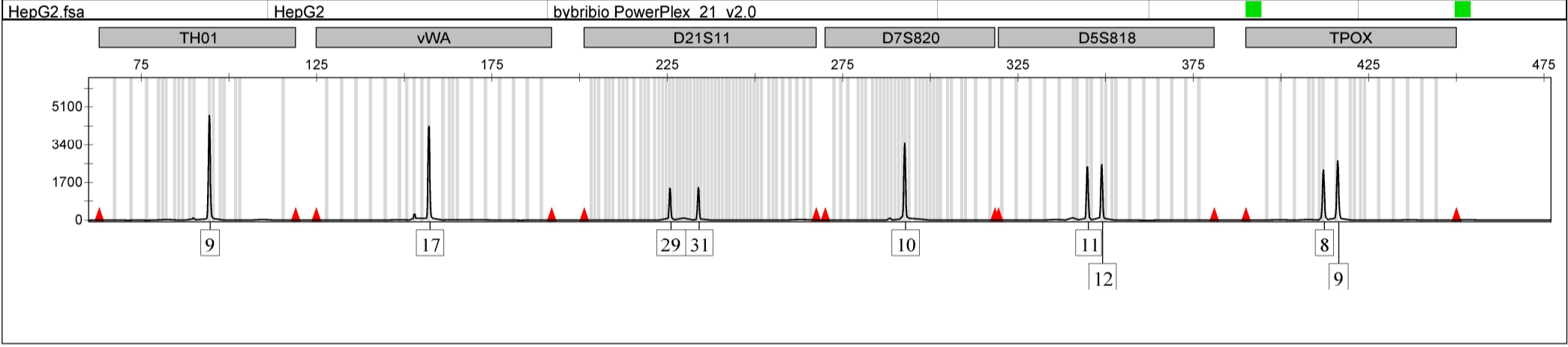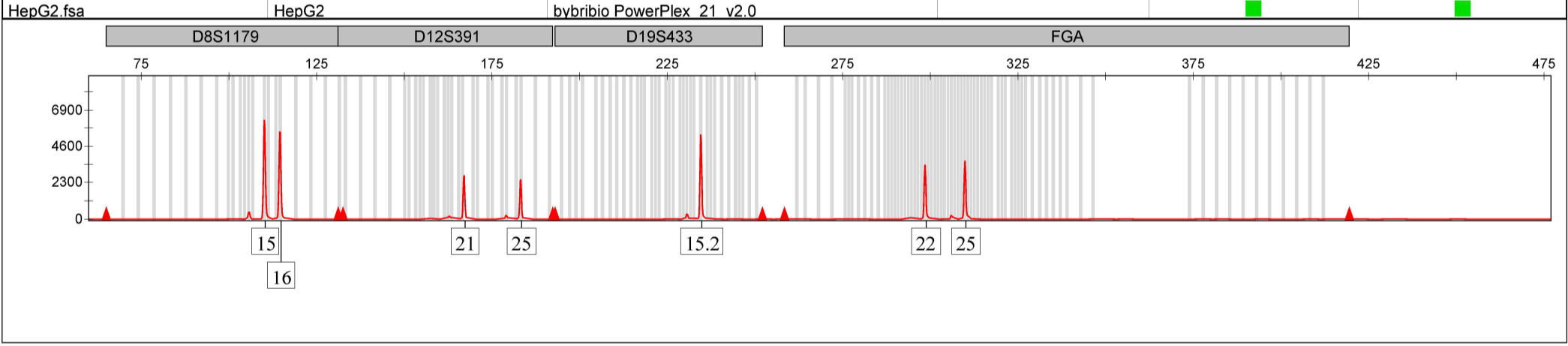

Supplement: S4 File — (ZIP) [file pone.0336879.s004.zip › STR typing map of HepG2/HePG2.pdf]
