# Supplementary material for: Expression of Concern: Natural borneol, a monoterpenoid compound, potentiates selenocystine-induced apoptosis in human hepatocellular carcinoma cells by enhancement of cellular uptake and activation of ROS-mediated DNA damage
Source: PLoS One. 2025 Dec 1;20(12):e0336879. doi: 10.1371/journal.pone.0336879 (PMC12668515; doi:10.1371/journal.pone.0336879)
Supplement: S2 File — (PPTX) [file pone.0336879.s002.pptx]

## Slide 1
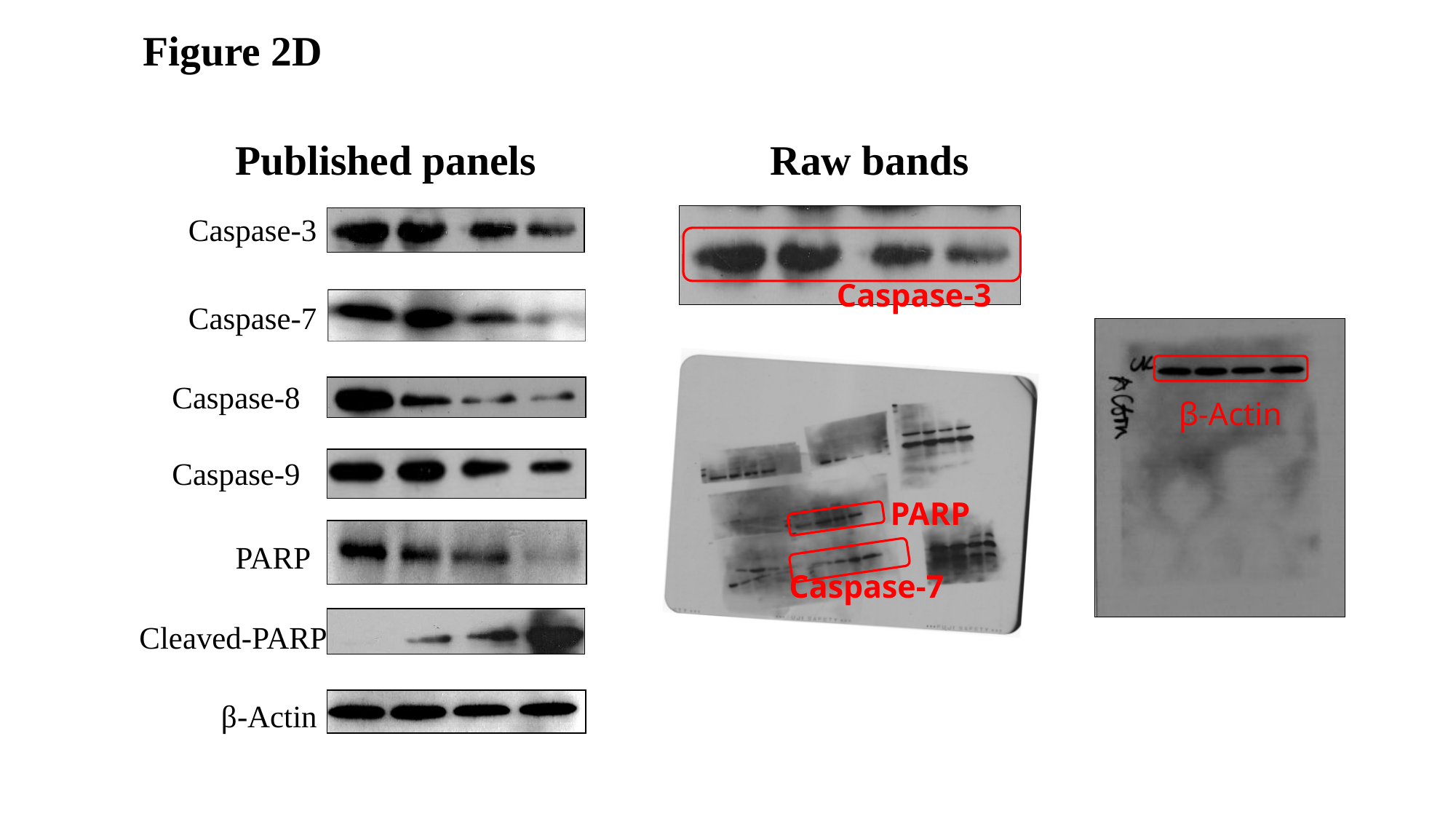

Figure 2D
Published panels
Raw bands
Caspase-3
Caspase-3
Caspase-7
Caspase-8
β-Actin
Caspase-9
PARP
PARP
Caspase-7
Cleaved-PARP
β-Actin

## Slide 2
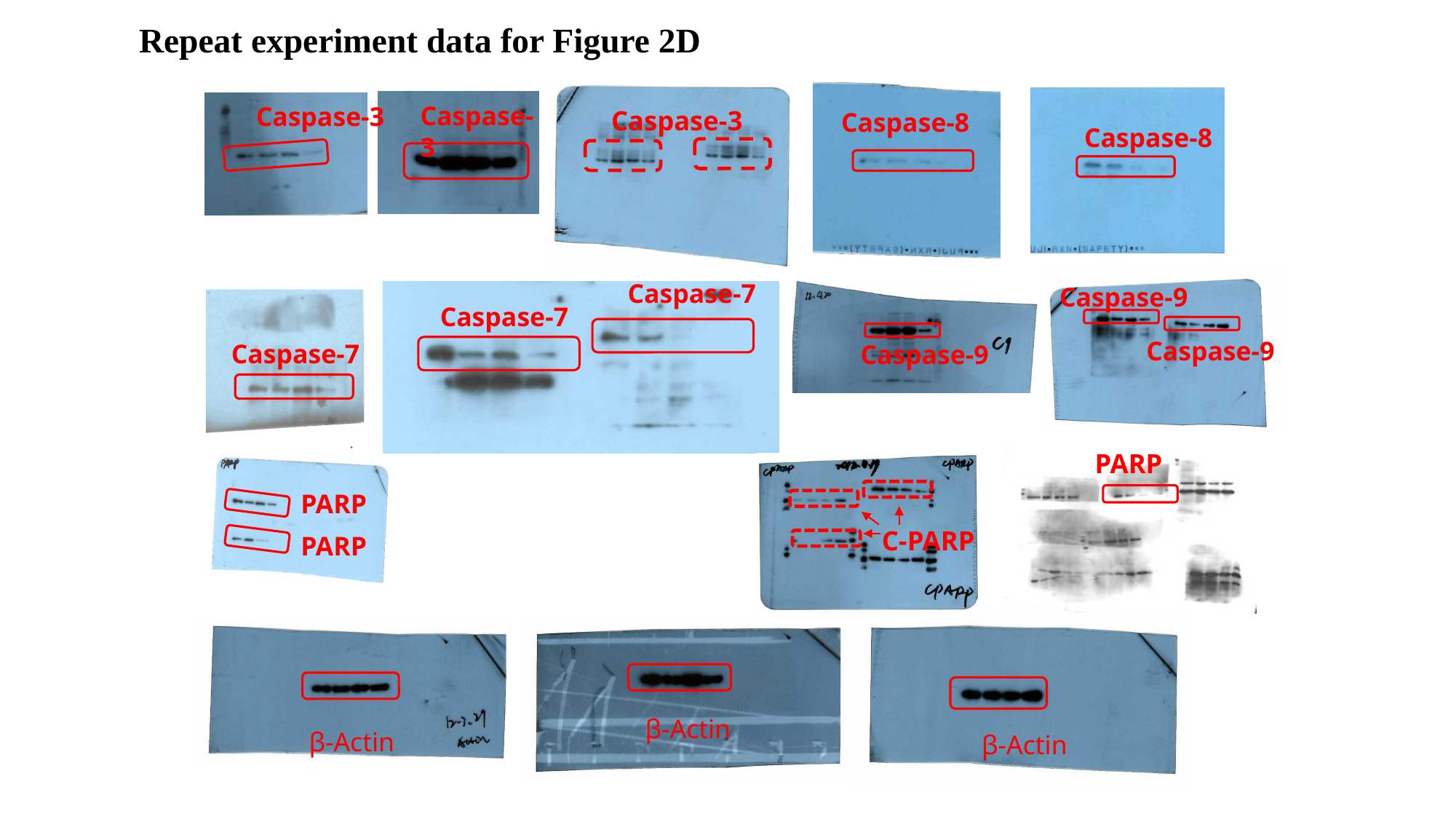

Repeat experiment data for Figure 2D
Caspase-8
Caspase-8
Caspase-3
Caspase-3
Caspase-3
Caspase-7
Caspase-7
Caspase-7
Caspase-9
Caspase-9
Caspase-9
PARP
PARP
PARP
C-PARP
β-Actin
β-Actin
β-Actin

## Slide 3
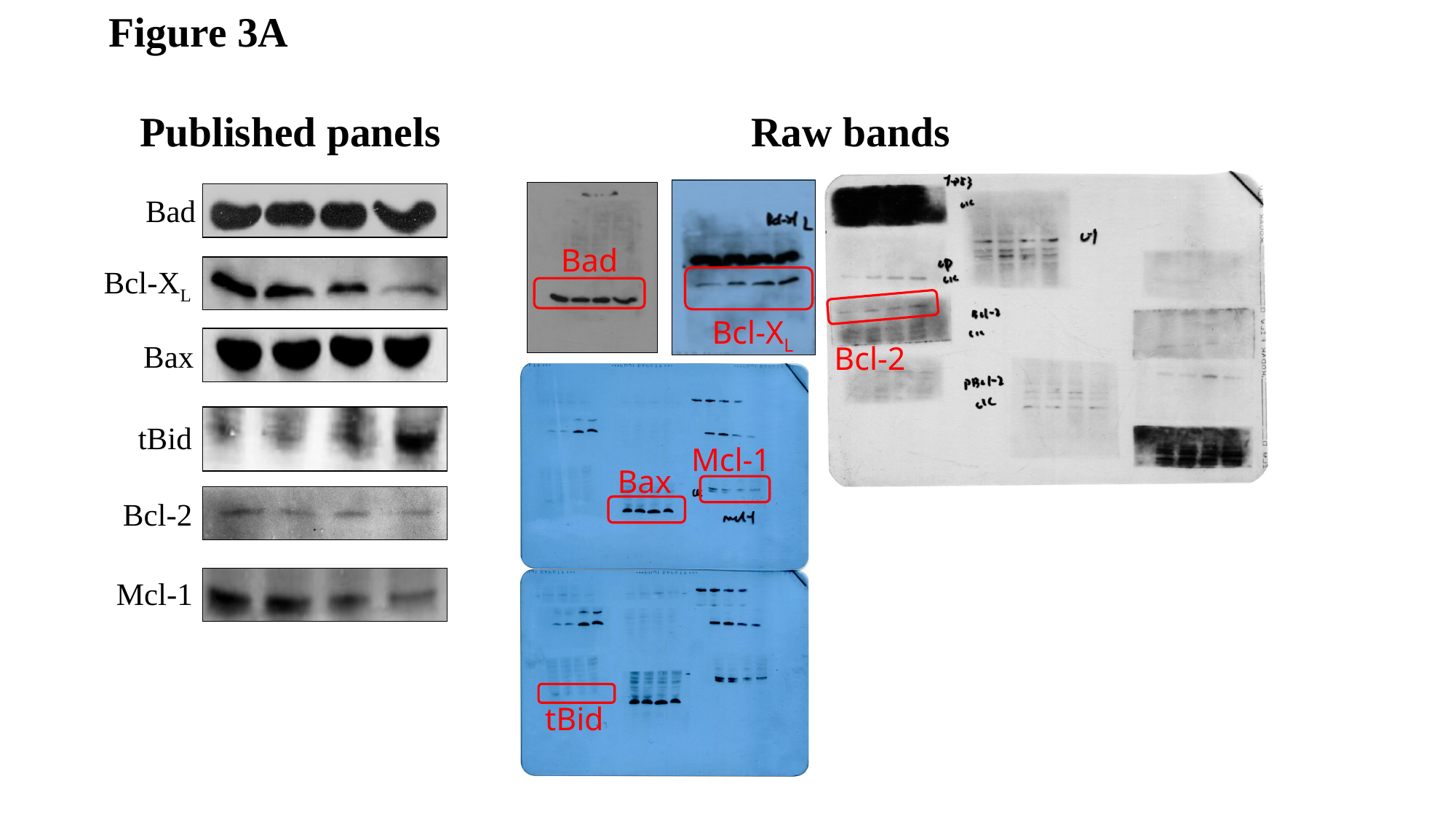

Figure 3A
Published panels
Raw bands
Bad
Bad
Bcl-XL
Bcl-XL
Bax
Bcl-2
tBid
Mcl-1
Bax
Bcl-2
Mcl-1
tBid

## Slide 4
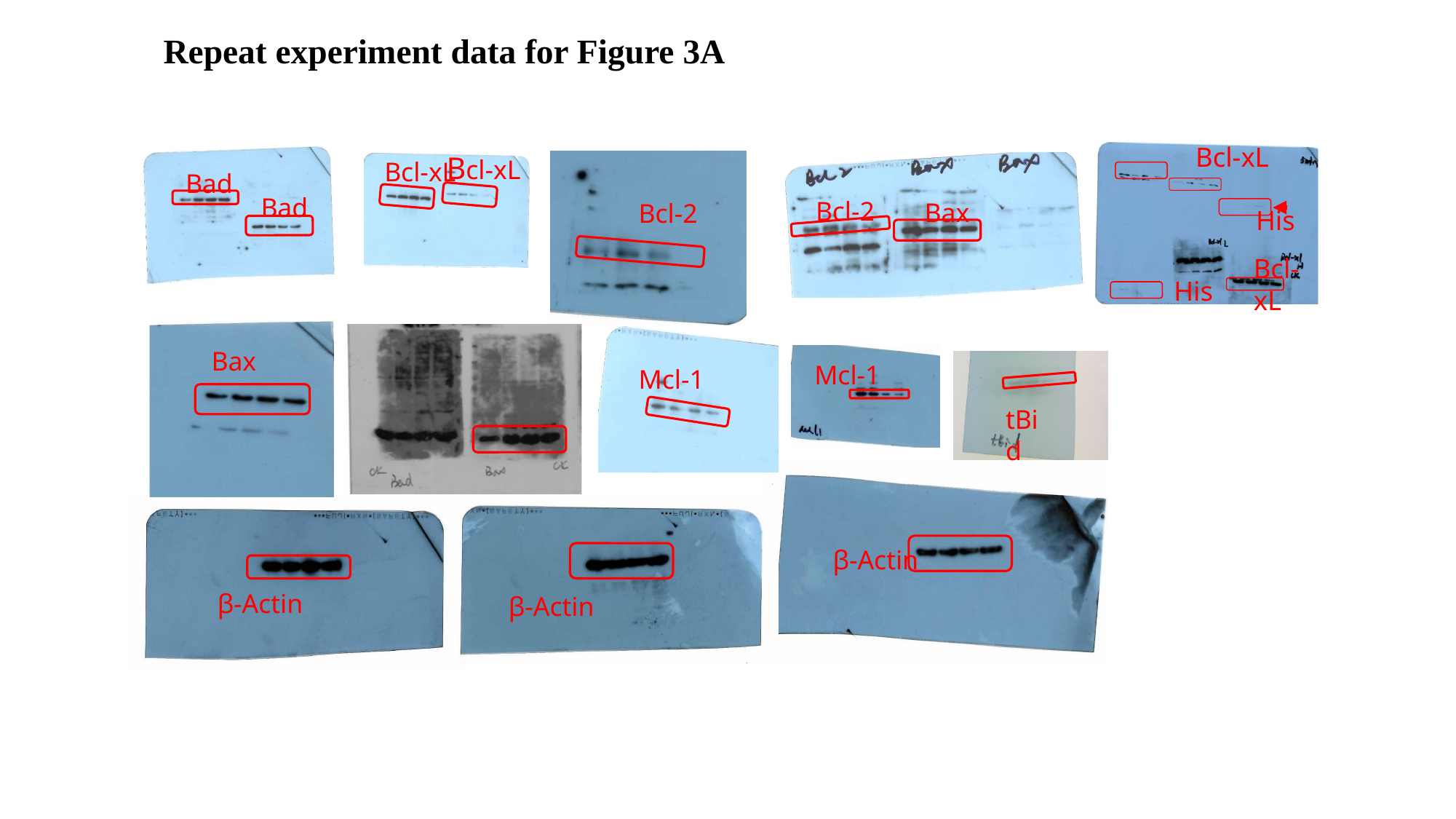

Repeat experiment data for Figure 3A
Bcl-xL
His
Bcl-xL
His
Bad
Bad
Bcl-xL
Bcl-xL
Bcl-2
Bcl-2
Bax
Mcl-1
Mcl-1
Bax
tBid
β-Actin
β-Actin
β-Actin

## Slide 5
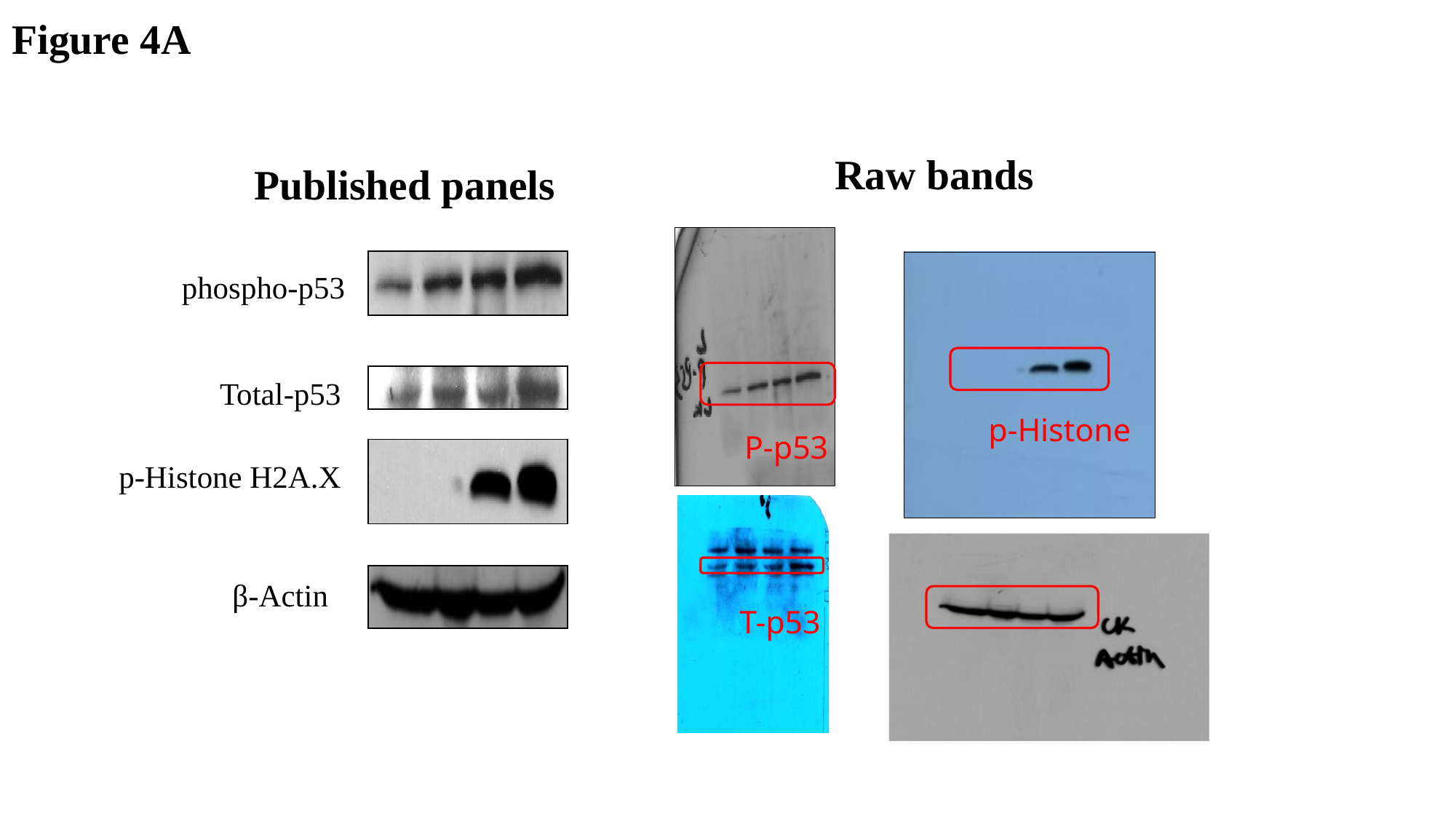

Figure 4A
Raw bands
Published panels
phospho-p53
Total-p53
p-Histone
P-p53
p-Histone H2A.X
β-Actin
T-p53

## Slide 6
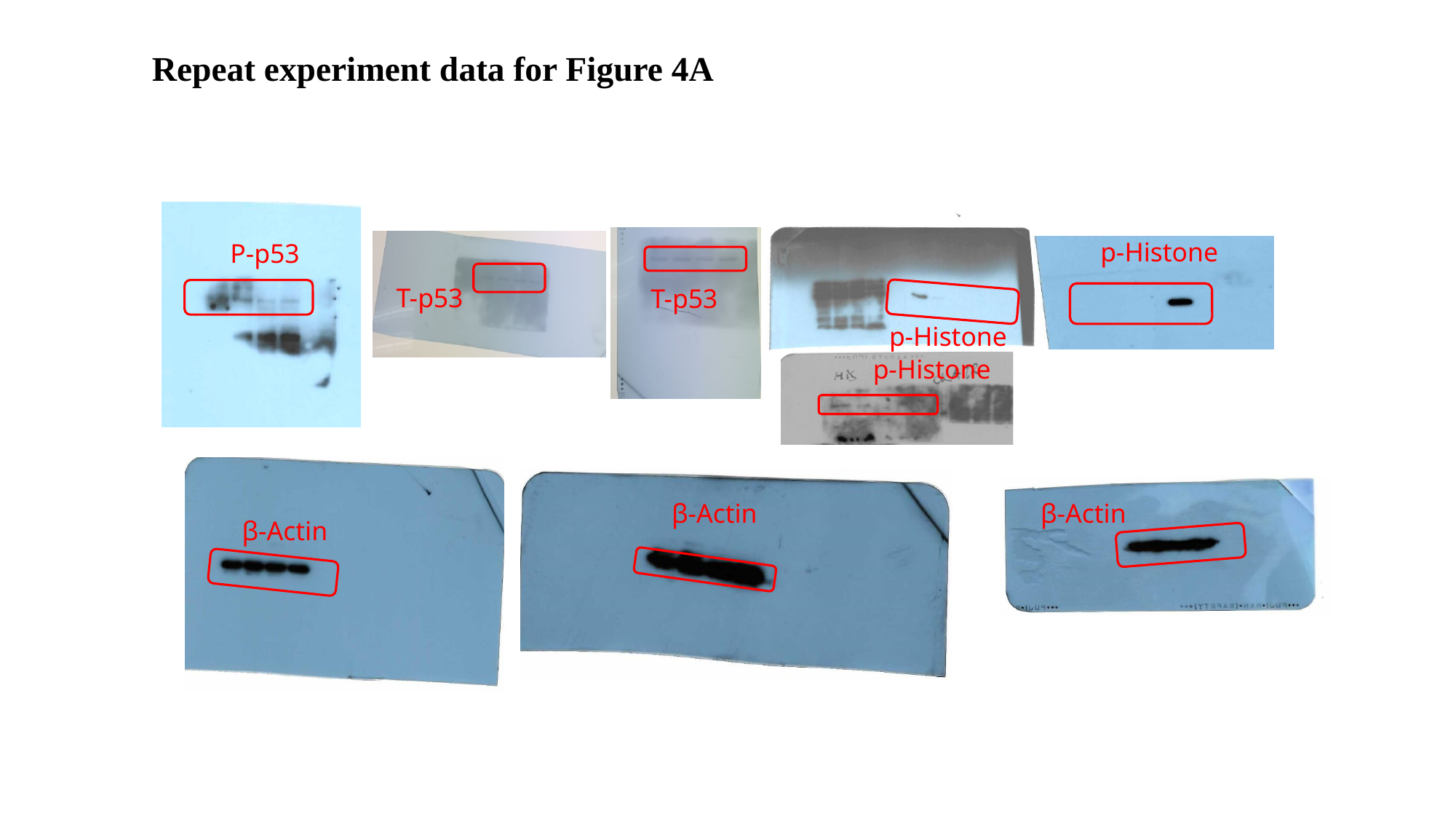

Repeat experiment data for Figure 4A
p-Histone
P-p53
T-p53
T-p53
p-Histone
p-Histone
β-Actin
β-Actin
β-Actin

## Slide 7
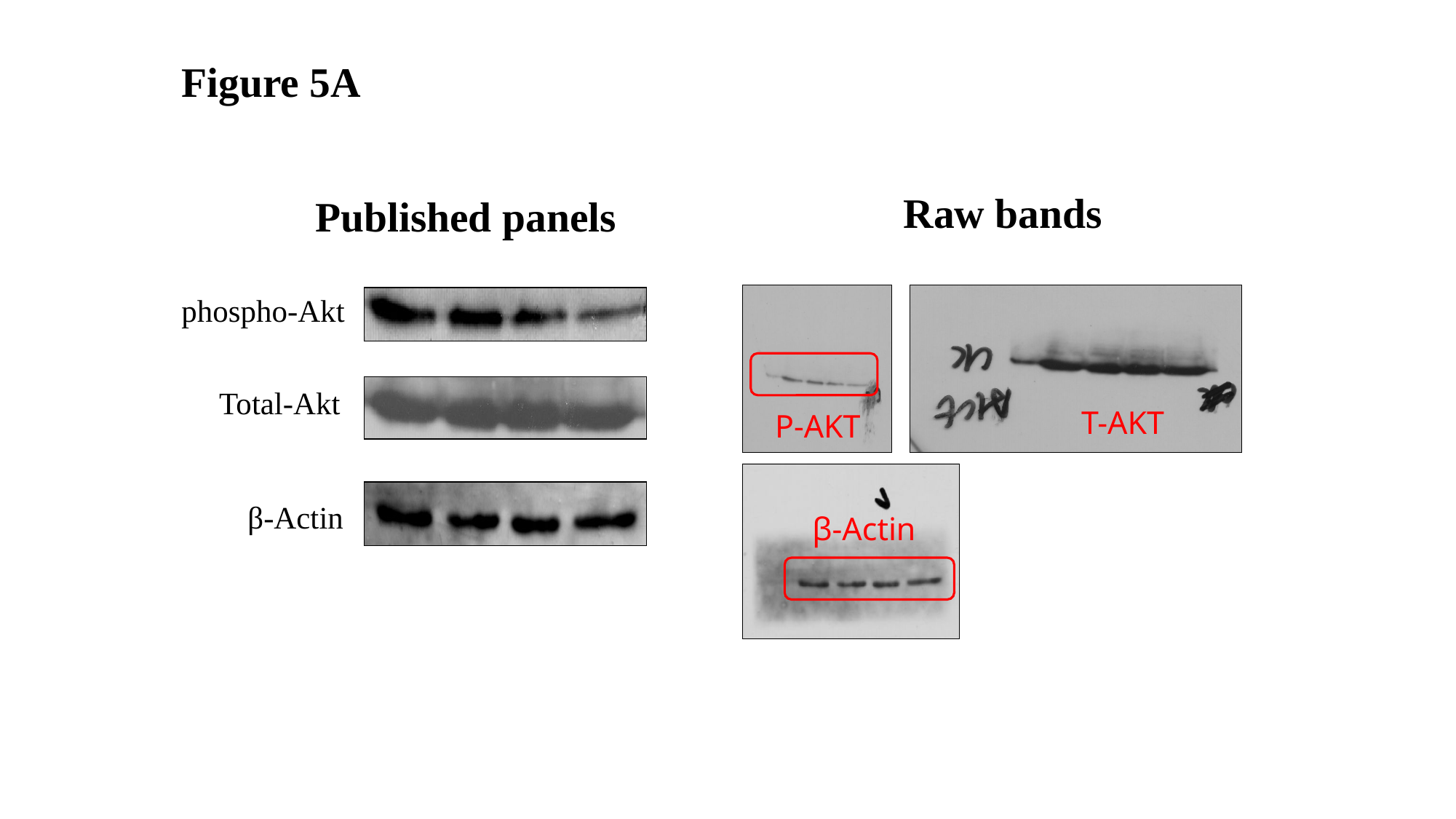

Figure 5A
Raw bands
Published panels
phospho-Akt
Total-Akt
T-AKT
P-AKT
β-Actin
β-Actin

## Slide 8
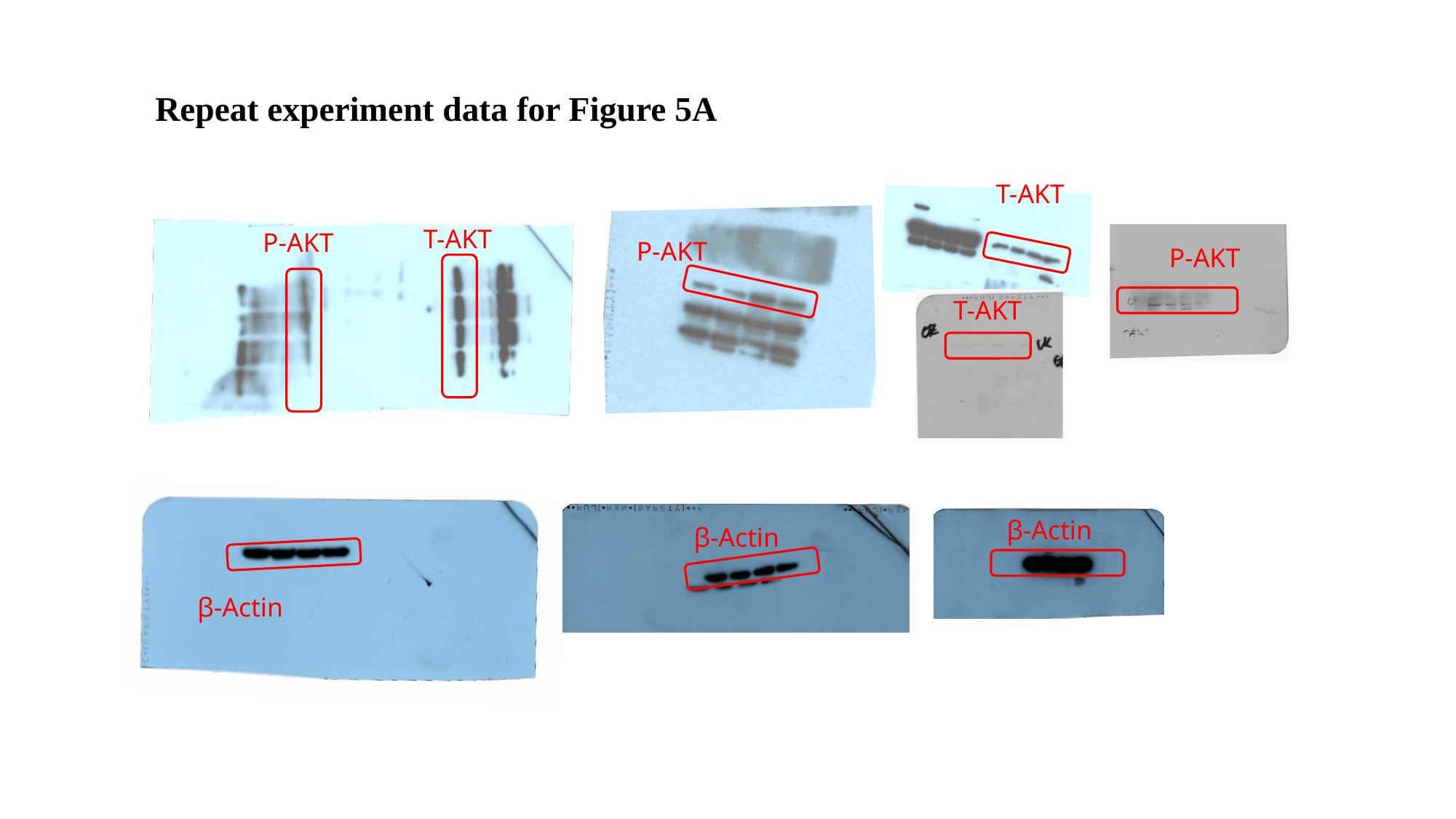

Repeat experiment data for Figure 5A
T-AKT
T-AKT
P-AKT
P-AKT
P-AKT
T-AKT
β-Actin
β-Actin
β-Actin

## Slide 9
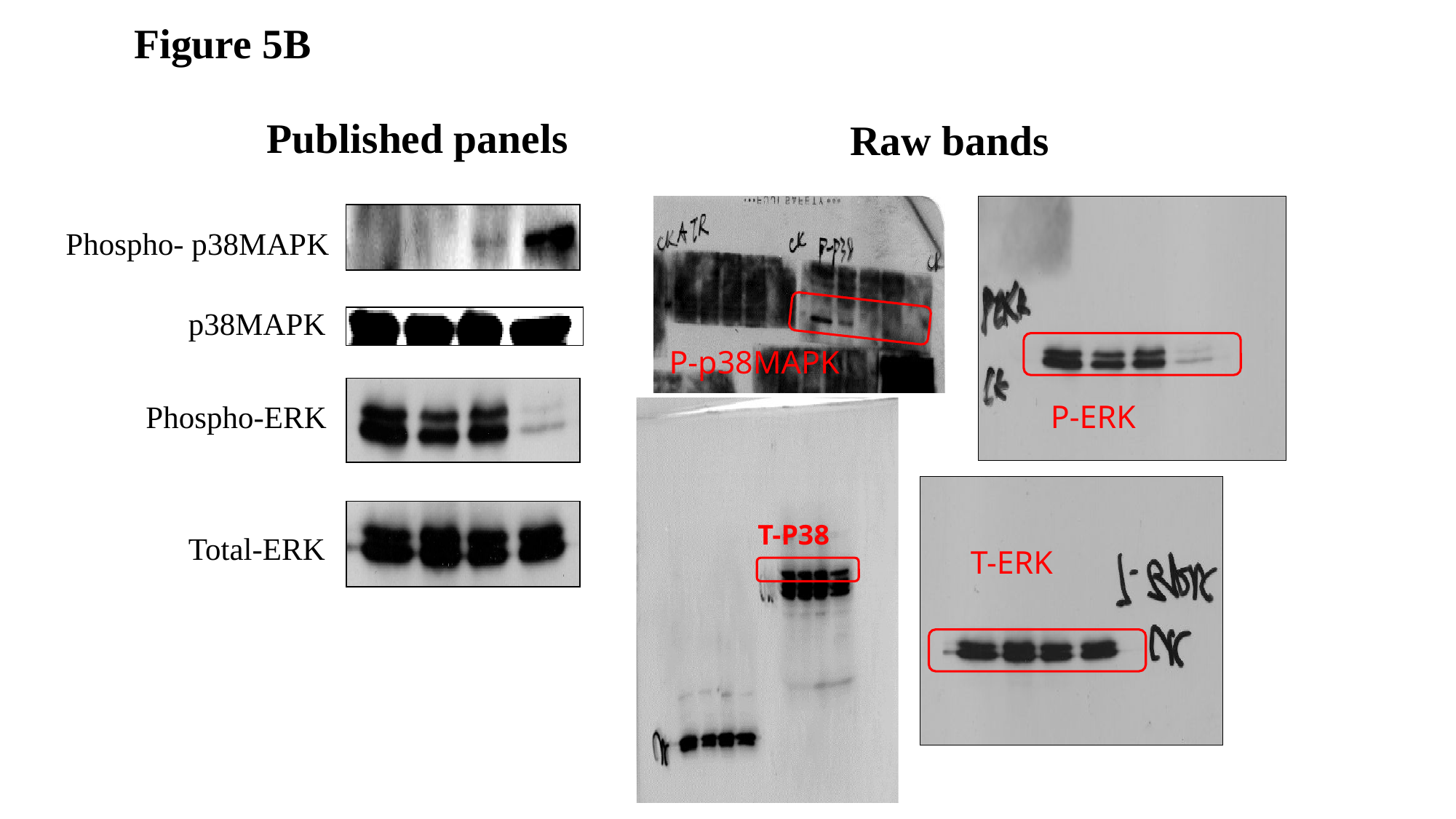

Figure 5B
Published panels
Raw bands
Phospho- p38MAPK
p38MAPK
P-p38MAPK
Phospho-ERK
P-ERK
T-P38
Total-ERK
T-ERK

## Slide 10
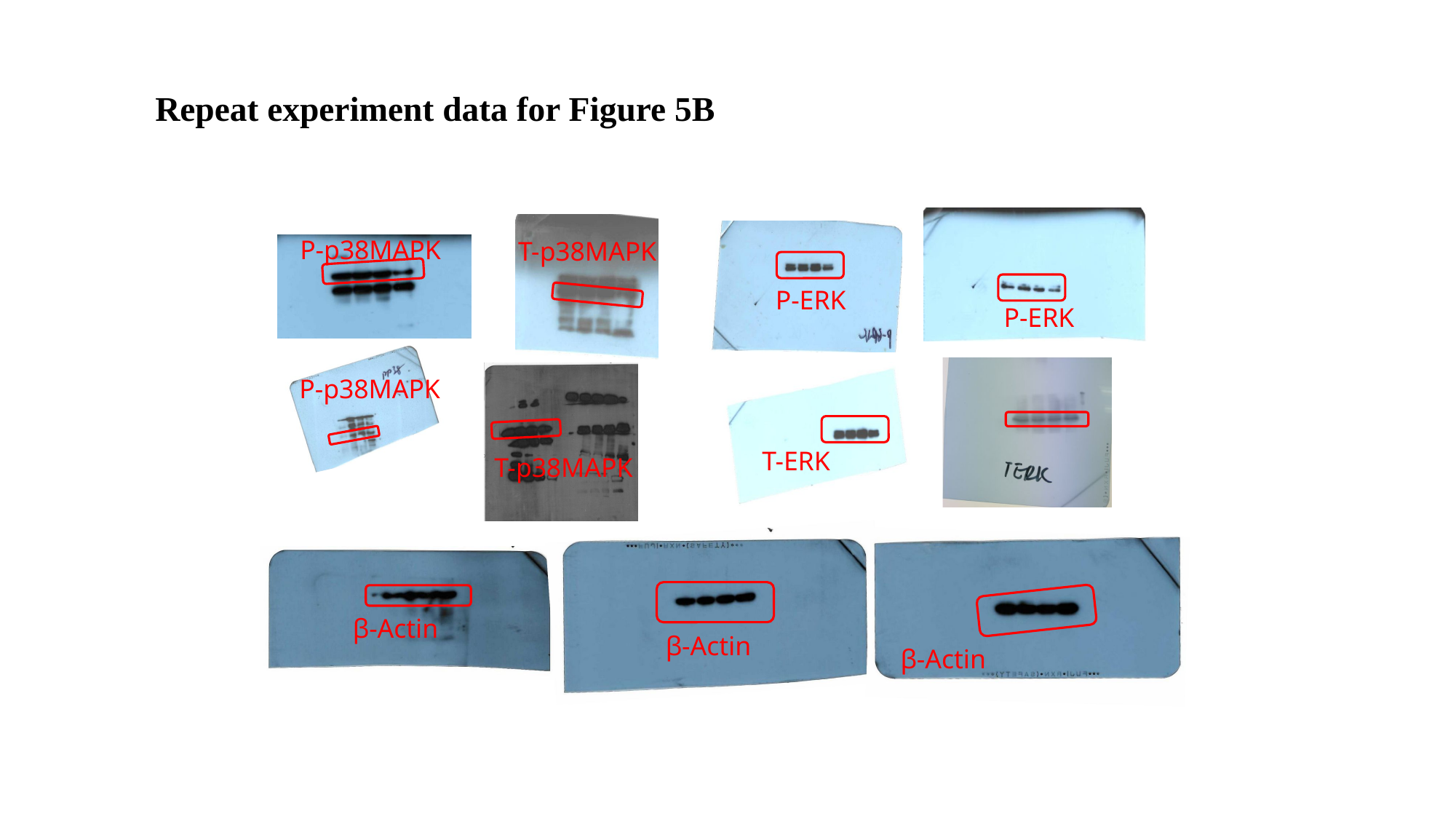

Repeat experiment data for Figure 5B
P-p38MAPK
T-p38MAPK
P-ERK
P-ERK
P-p38MAPK
T-ERK
T-p38MAPK
β-Actin
β-Actin
β-Actin
