# Supplementary material for: Expression of Concern: Natural borneol, a monoterpenoid compound, potentiates selenocystine-induced apoptosis in human hepatocellular carcinoma cells by enhancement of cellular uptake and activation of ROS-mediated DNA damage
Source: PLoS One. 2025 Dec 1;20(12):e0336879. doi: 10.1371/journal.pone.0336879 (PMC12668515; doi:10.1371/journal.pone.0336879)
Supplement: S3 File — (ZIP) [file pone.0336879.s003.zip › Fig 2E .pptx]

## Slide 1
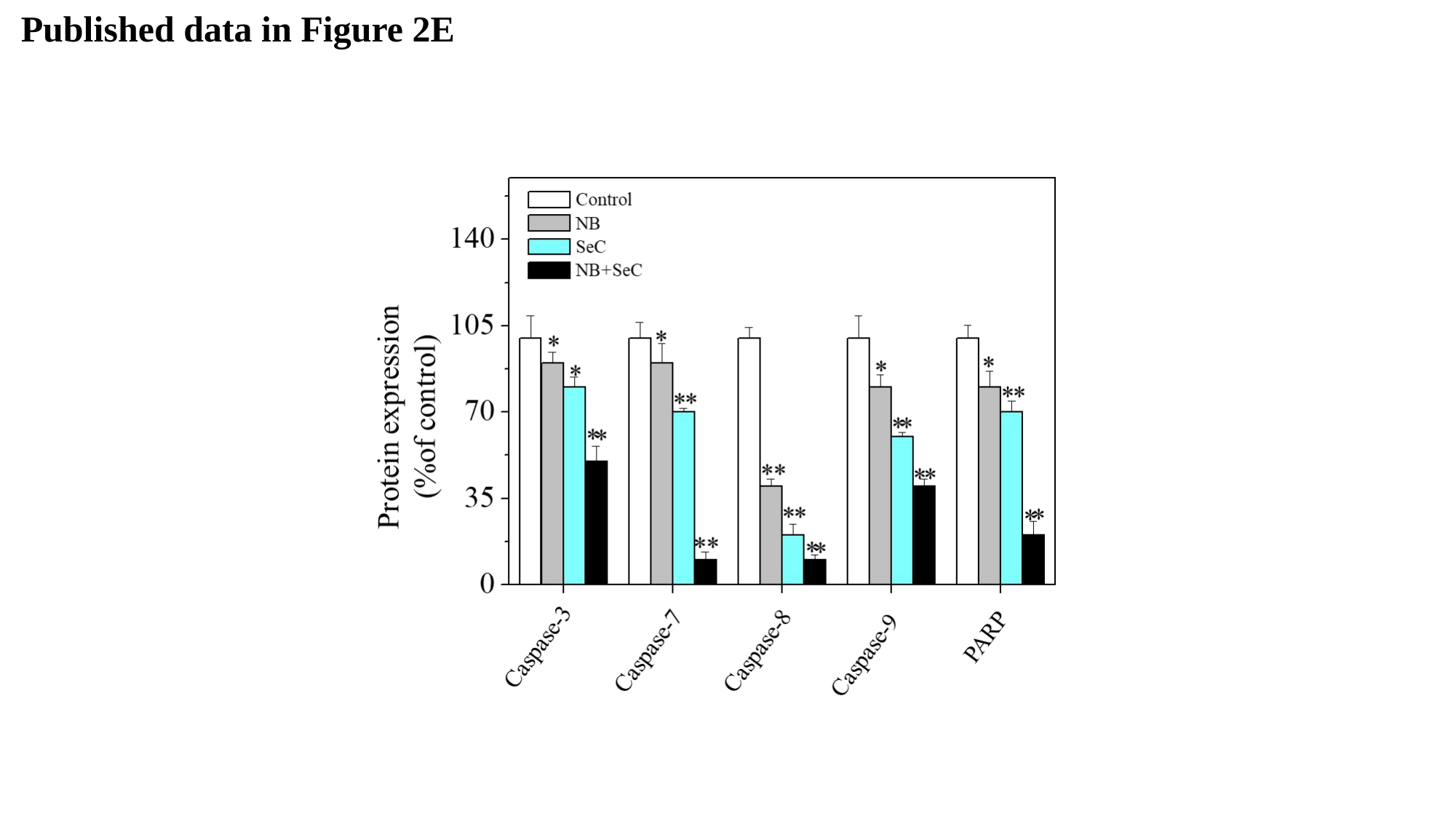

Published data in Figure 2E

## Slide 2
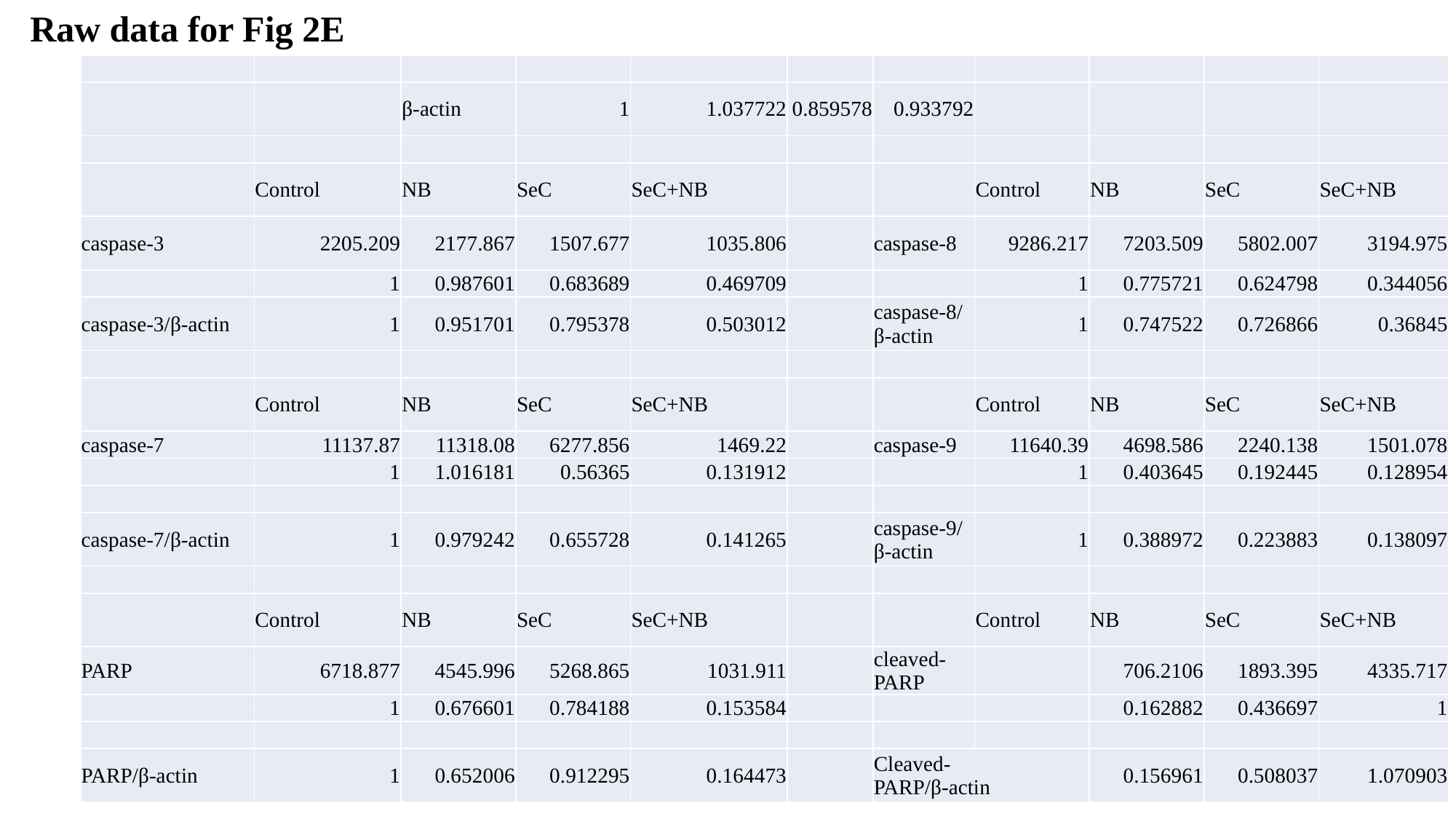

Raw data for Fig 2E
| | | | | | | | | | | |
| --- | --- | --- | --- | --- | --- | --- | --- | --- | --- | --- |
| | | β-actin | 1 | 1.037722 | 0.859578 | 0.933792 | | | | |
| | | | | | | | | | | |
| | Control | NB | SeC | SeC+NB | | | Control | NB | SeC | SeC+NB |
| caspase-3 | 2205.209 | 2177.867 | 1507.677 | 1035.806 | | caspase-8 | 9286.217 | 7203.509 | 5802.007 | 3194.975 |
| | 1 | 0.987601 | 0.683689 | 0.469709 | | | 1 | 0.775721 | 0.624798 | 0.344056 |
| caspase-3/β-actin | 1 | 0.951701 | 0.795378 | 0.503012 | | caspase-8/β-actin | 1 | 0.747522 | 0.726866 | 0.36845 |
| | | | | | | | | | | |
| | Control | NB | SeC | SeC+NB | | | Control | NB | SeC | SeC+NB |
| caspase-7 | 11137.87 | 11318.08 | 6277.856 | 1469.22 | | caspase-9 | 11640.39 | 4698.586 | 2240.138 | 1501.078 |
| | 1 | 1.016181 | 0.56365 | 0.131912 | | | 1 | 0.403645 | 0.192445 | 0.128954 |
| | | | | | | | | | | |
| caspase-7/β-actin | 1 | 0.979242 | 0.655728 | 0.141265 | | caspase-9/β-actin | 1 | 0.388972 | 0.223883 | 0.138097 |
| | | | | | | | | | | |
| | Control | NB | SeC | SeC+NB | | | Control | NB | SeC | SeC+NB |
| PARP | 6718.877 | 4545.996 | 5268.865 | 1031.911 | | cleaved-PARP | | 706.2106 | 1893.395 | 4335.717 |
| | 1 | 0.676601 | 0.784188 | 0.153584 | | | | 0.162882 | 0.436697 | 1 |
| | | | | | | | | | | |
| PARP/β-actin | 1 | 0.652006 | 0.912295 | 0.164473 | | Cleaved- PARP/β-actin | | 0.156961 | 0.508037 | 1.070903 |
