# Supplementary material for: Expression of Concern: Natural borneol, a monoterpenoid compound, potentiates selenocystine-induced apoptosis in human hepatocellular carcinoma cells by enhancement of cellular uptake and activation of ROS-mediated DNA damage
Source: PLoS One. 2025 Dec 1;20(12):e0336879. doi: 10.1371/journal.pone.0336879 (PMC12668515; doi:10.1371/journal.pone.0336879)
Supplement: S3 File — (ZIP) [file pone.0336879.s003.zip › Fig 3B.pptx]

## Slide 1
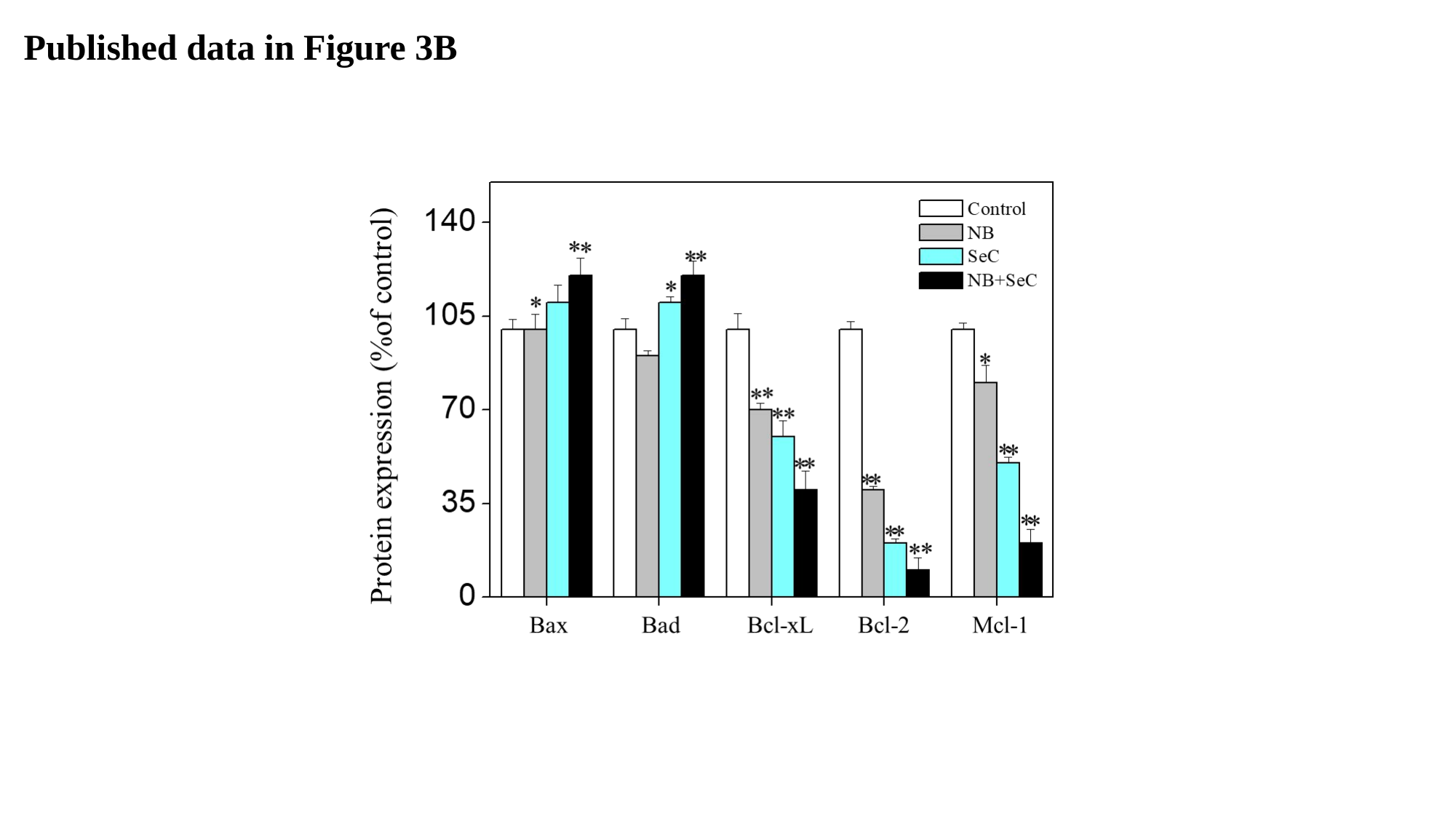

Published data in Figure 3B

## Slide 2
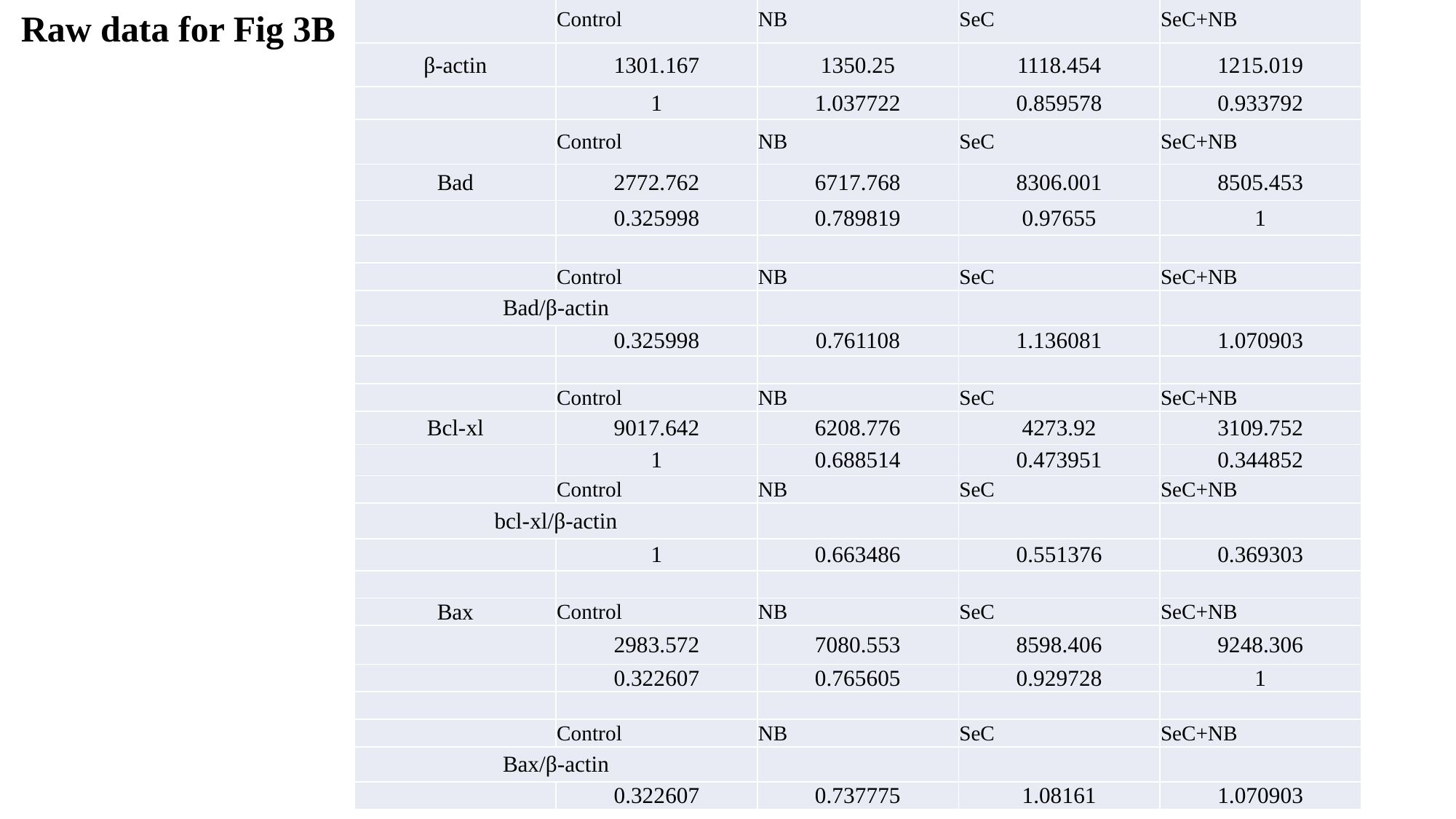

Raw data for Fig 3B
| | Control | NB | SeC | SeC+NB |
| --- | --- | --- | --- | --- |
| β-actin | 1301.167 | 1350.25 | 1118.454 | 1215.019 |
| | 1 | 1.037722 | 0.859578 | 0.933792 |
| | Control | NB | SeC | SeC+NB |
| Bad | 2772.762 | 6717.768 | 8306.001 | 8505.453 |
| | 0.325998 | 0.789819 | 0.97655 | 1 |
| | | | | |
| | Control | NB | SeC | SeC+NB |
| Bad/β-actin | | | | |
| | 0.325998 | 0.761108 | 1.136081 | 1.070903 |
| | | | | |
| | Control | NB | SeC | SeC+NB |
| Bcl-xl | 9017.642 | 6208.776 | 4273.92 | 3109.752 |
| | 1 | 0.688514 | 0.473951 | 0.344852 |
| | Control | NB | SeC | SeC+NB |
| bcl-xl/β-actin | | | | |
| | 1 | 0.663486 | 0.551376 | 0.369303 |
| | | | | |
| Bax | Control | NB | SeC | SeC+NB |
| | 2983.572 | 7080.553 | 8598.406 | 9248.306 |
| | 0.322607 | 0.765605 | 0.929728 | 1 |
| | | | | |
| | Control | NB | SeC | SeC+NB |
| Bax/β-actin | | | | |
| | 0.322607 | 0.737775 | 1.08161 | 1.070903 |
