# Supplementary material for: Expression of Concern: Natural borneol, a monoterpenoid compound, potentiates selenocystine-induced apoptosis in human hepatocellular carcinoma cells by enhancement of cellular uptake and activation of ROS-mediated DNA damage
Source: PLoS One. 2025 Dec 1;20(12):e0336879. doi: 10.1371/journal.pone.0336879 (PMC12668515; doi:10.1371/journal.pone.0336879)
Supplement: S3 File — (ZIP) [file pone.0336879.s003.zip › Fig 5C .pptx]

## Slide 1
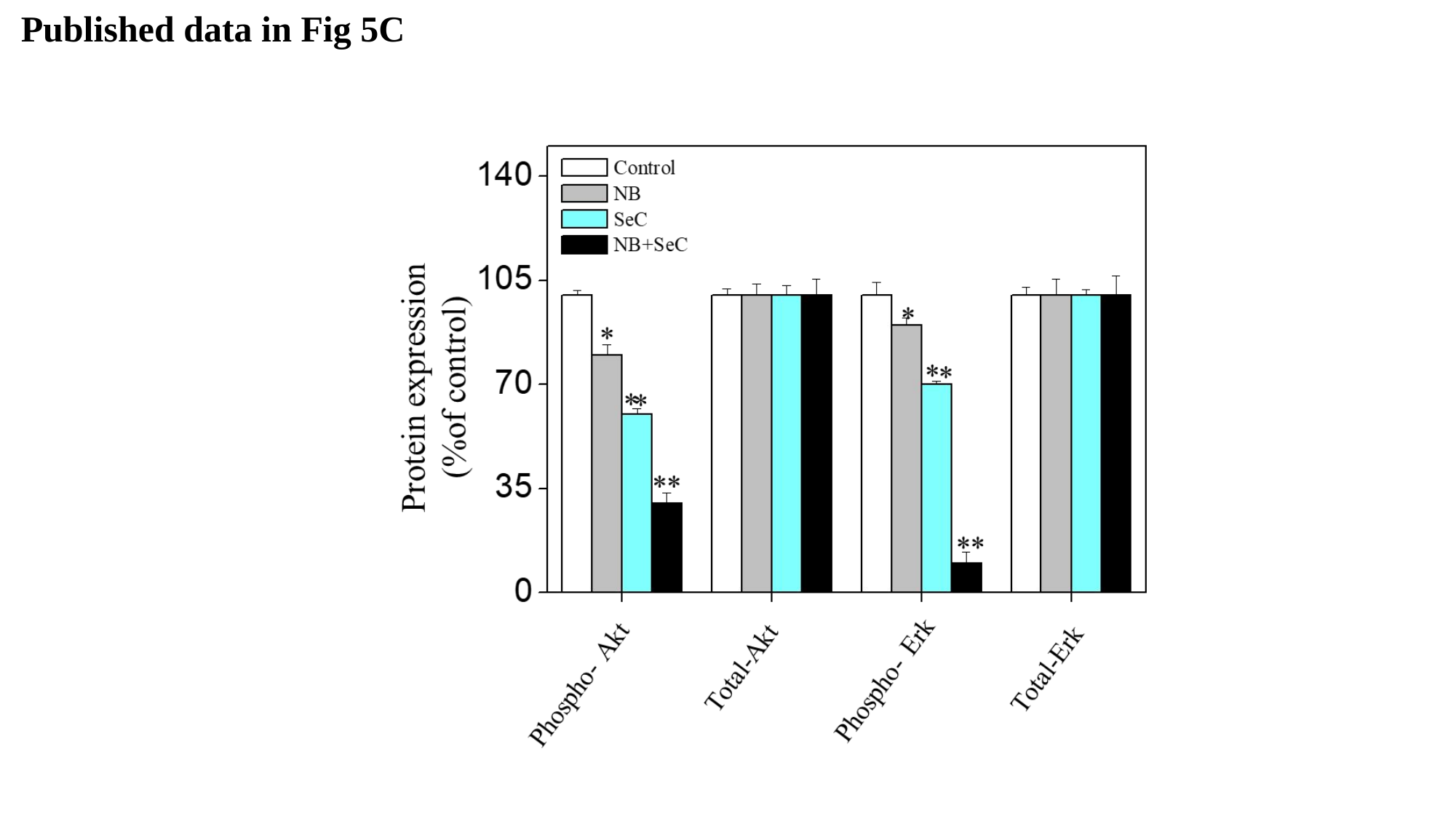

Published data in Fig 5C

## Slide 2
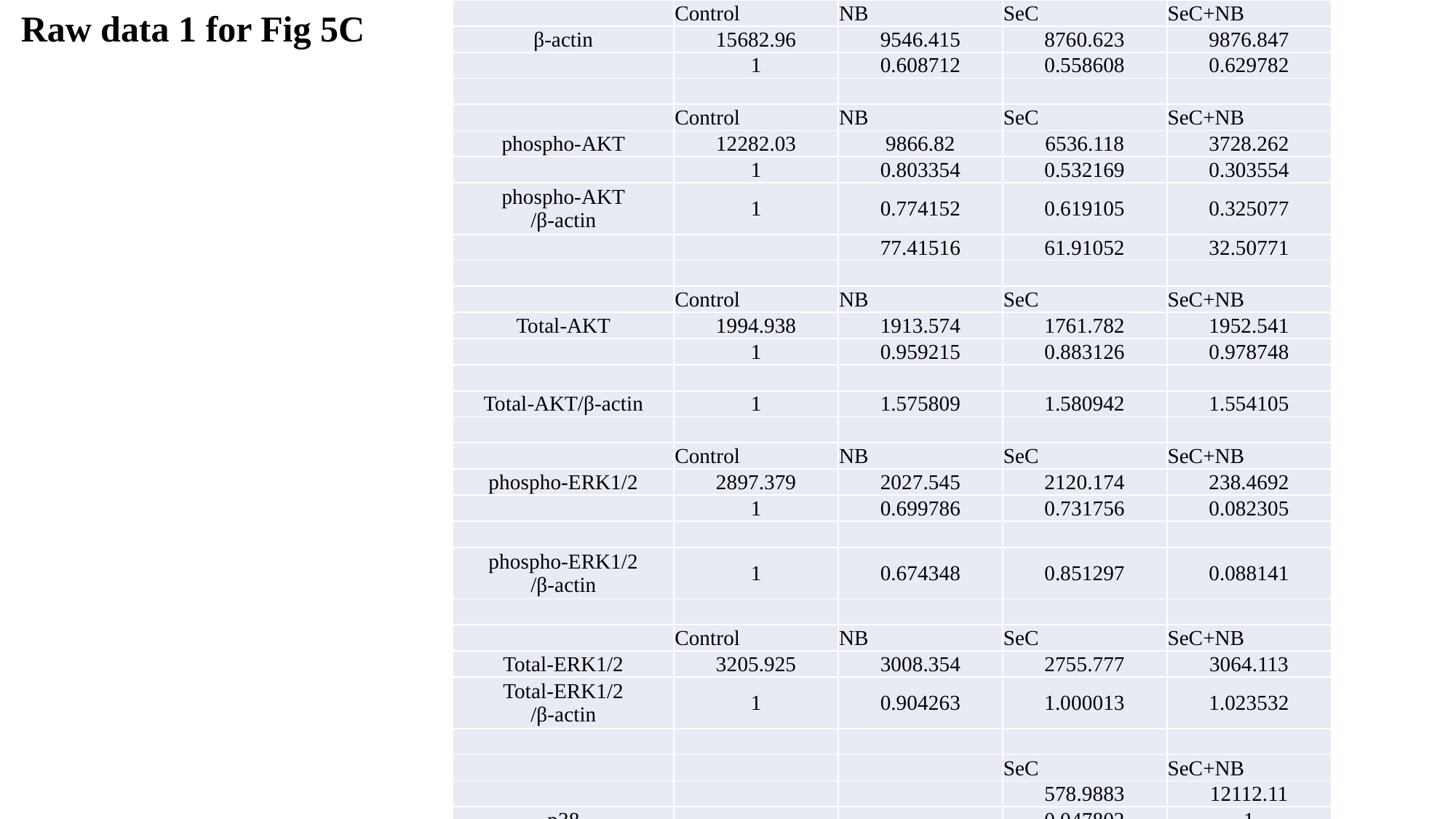

Raw data 1 for Fig 5C
| | Control | NB | SeC | SeC+NB |
| --- | --- | --- | --- | --- |
| β-actin | 15682.96 | 9546.415 | 8760.623 | 9876.847 |
| | 1 | 0.608712 | 0.558608 | 0.629782 |
| | | | | |
| | Control | NB | SeC | SeC+NB |
| phospho-AKT | 12282.03 | 9866.82 | 6536.118 | 3728.262 |
| | 1 | 0.803354 | 0.532169 | 0.303554 |
| phospho-AKT /β-actin | 1 | 0.774152 | 0.619105 | 0.325077 |
| | | 77.41516 | 61.91052 | 32.50771 |
| | | | | |
| | Control | NB | SeC | SeC+NB |
| Total-AKT | 1994.938 | 1913.574 | 1761.782 | 1952.541 |
| | 1 | 0.959215 | 0.883126 | 0.978748 |
| | | | | |
| Total-AKT/β-actin | 1 | 1.575809 | 1.580942 | 1.554105 |
| | | | | |
| | Control | NB | SeC | SeC+NB |
| phospho-ERK1/2 | 2897.379 | 2027.545 | 2120.174 | 238.4692 |
| | 1 | 0.699786 | 0.731756 | 0.082305 |
| | | | | |
| phospho-ERK1/2 /β-actin | 1 | 0.674348 | 0.851297 | 0.088141 |
| | | | | |
| | Control | NB | SeC | SeC+NB |
| Total-ERK1/2 | 3205.925 | 3008.354 | 2755.777 | 3064.113 |
| Total-ERK1/2 /β-actin | 1 | 0.904263 | 1.000013 | 1.023532 |
| | | | | |
| | | | SeC | SeC+NB |
| | | | 578.9883 | 12112.11 |
| p38 | | | 0.047802 | 1 |
| p38/β-actin | | | 0.055612 | 1.070903 |
