# Supplementary material for: Expression of Concern: Natural borneol, a monoterpenoid compound, potentiates selenocystine-induced apoptosis in human hepatocellular carcinoma cells by enhancement of cellular uptake and activation of ROS-mediated DNA damage
Source: PLoS One. 2025 Dec 1;20(12):e0336879. doi: 10.1371/journal.pone.0336879 (PMC12668515; doi:10.1371/journal.pone.0336879)
Supplement: S4 File — (ZIP) [file pone.0336879.s004.zip › Report of HepG2 cell Authentication(KPSC20200).pdf]

Cell Line Authentication Service
STR Profile Report

Sample Submitted By: Jinan University

Email Address: 1296902957@qq.com

Hybribio Sales Order: KPSC20200

Cell Line Designation: HepG2

Date Sample Received: 07/07/2022

Report Date: 07/08/2022

Methodology:

Twenty short tandem repeat (STR) loci plus the gender determining locus, Amelogenin, were amplified using a commercially available STR profiling Kit. The cell line sample was processed using the ABI Prism® 3500XL Genetic Analyzer. Data were analyzed using GeneMapper® 5 software (Applied Biosystems). Appropriate positive and negative controls were run and confirmed for each sample submitted.

Data Interpretation:

Cell lines were authenticated using Short Tandem Repeat (STR) analysis as described in 2012 in ANSI Standard (ANSI/ATCC ASN-0002-2011 *Authentication of Human Cell Lines: Standardization of STR Profiling*) by the ATCC Standards Development Organization (SDO).

Test Results of Submitted Sample:

Alleles / STR Loci	Allele 1	Allele 2	Allele 3	Allele 4
**Amelogenin**	X	Y		
**D3S1358**	15	16		
**D1S1656**	11	12		
**D6S1043**	13	13		
**D13S317**	9	13		
**PentaE**	15	20		
**D16S539**	12	12		
**D18S51**	13	14		
**D2S1338**	19	20		
**CSF1PO**	10	11		
**PentaD**	9	13		
**TH01**	9	9		
**vWA**	17	17		
**D21S11**	29	31		
**D7S820**	10	10		
**D5S818**	11	12		
**TPOX**	8	9		
**D8S1179**	15	16		
**D12S391**	21	25		
**D19S433**	15.2	15.2		
**FGA**	22	25		

NOTE:

Ⅰ. Loci highlighted in grey (8 core STR loci plus Amelogenin) can be made public to verify cell identity. In order to protect the identity of the donor, Please do not publish the allele calls from the STR loci tested.

Ⅱ. A relatively common occurrence of STR typing of human cancer cell lines is multiple Alleles at several loci. Three or more Alleles at one or two loci may be due to somatic mutation, trisomy or gene duplications. Events with more than three Alleles at more than three loci may be due to cellular contamination.

Ⅲ. Electropherograms showing raw data and Map are attached.

Result of searching against Hybribio STR Profile Database:

Hybribio STR Searcher

Search by Amelogenin (AMEL) + 8loci:

Names	D5S818	D13S317	D7S820	D16S539	vWA	TH01	AM	TPOX	CSF1PO
Values	11,12	9,13	10,10	12,12	17,17	9,9	X,Y	8,9	10,11

Options

Matches>=80% Matches>=56% Matches>=0% Another Algorithm Export Exit

Matches:

NO.	Percent Match	Cell No.	Cell name	D5S818	D13S317	D7S820	D16S539	vWA	TH01	AM	TPOX	CSF1PO
			Query(Your Cell)	11,12	9,13	10,10	12,12	17,17	9,9	X,Y	8,9	10,11
1	100%	MRA-975	HC-04	11,12	9,13	10,10	12,12	17,17	9,9	X,Y	8,9	10,11
2	100%	RCB1681	GS-HepG2	11,12	9,13	10,10	12,12	17,17	9,9	X,Y	8,9	10,11
3	93%	RCB1648	Hep G2	11,12	9,13	10,10	12,13	17,17	9,9	X,Y	8,9	10,11
4	93%	180	HEP-G2	11,12	9,13	10,10	12,13	17,17	9,9	X,Y	8,9	10,11
5	93%	CRC98	HepG2	11,12	9,13	10,10	12,13	17,17	9,9	X,Y	8,9	10,11
6	93%	HB-8065.1	HepG2/SF	11,12	9,13	10,10	12,13	17,17	9,9	X,Y	8,9	10,11
7	93%	CRL-11997	HEP G2/2.2.1	11,12	9,13	10,10	12,13	17,17	9,9	X,Y	8,9	10,11
8	93%	HB-8065	Hep G2	11,12	9,13	10,10	12,13	17,17	9,9	X,Y	8,9	10,11
9	93%	RCB1886	Hep G2	11,12	9,13	10,10	12,13	17,17	9,9	X,Y	8,9	10,11
10	93%	JCRB1054	Hep G2	11,12	9,13	10,10	12,13	17,17	9,9	X,Y	8,9	10,11
11	93%		Hep G2	11,12	9,13	10,10	12,13	17,17	9,9	X,Y	8,9	10,11
12	86%		C3A	11,13	9,13	10,10	12,13	17,17	9,9	X,Y	8,9	10,11
13	86%	CRL-10741	C3A [HepG2/C3A, derivative of Hep G2 (ATCC HB-8065)]	11,13	9,13	10,10	12,13	17,17	9,9	X,Y	8,9	10,11
14	81%	Cliff210	OVCAR-3	11,12	12,12	10,10	12,12	17,17	9,9	X,X	8,8	11,12

NOTE:

Ⅰ. The allele match algorithm compares the 8 core loci plus amelogenin ("AM" for short) only.

Ⅱ. The Hybribio STR Profile Database, which contain more than 8,000 human cell lines' STR profile from Cell Banks, i.e., ATCC, DSMZ, JCRB and RIKEN, and some other publications, is established by Guangdong Hybribio Biotech Ltd.

Explanation of Test Results:

Cell lines with ≥80% match are considered to be related, i.e., derived from a common ancestry. Cell lines with between a 55% to 80% match require further profiling for authentication of relatedness. Here only show cell lines with ≥80% match.

Contact Us:

Website：http://www.hybribio.cn/
Telephone：（020）34072008
E-mail：StemCell@hybribio.cn
Fax：（020）34072005

e-Signature, Technician: Lulian Wu 07/08/2022

e-Signature, Reviewer: Yan Zheng 07/08/2022

Addendum: Electropherogram (peak data) for Submitted Sample

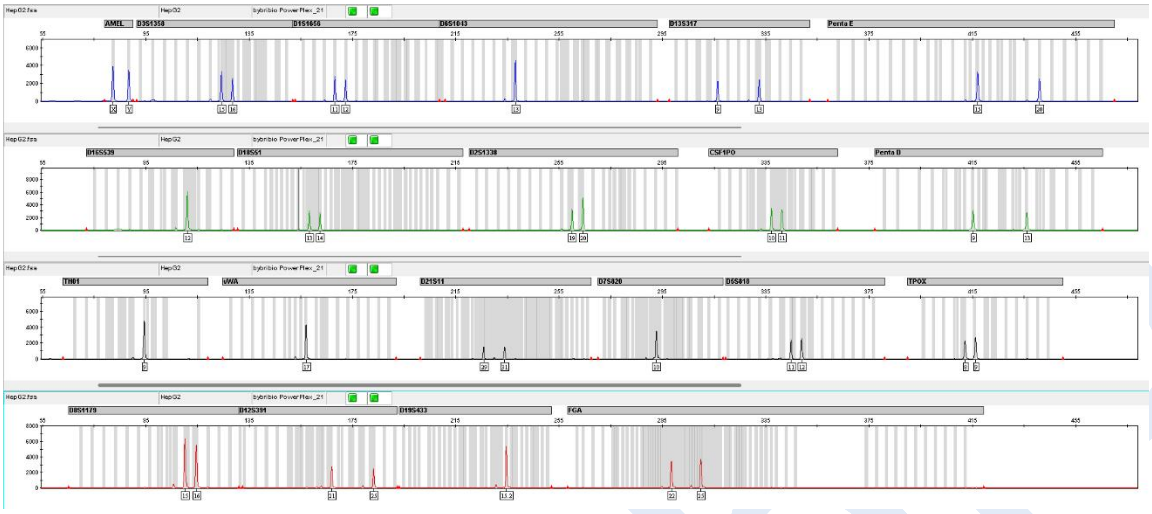

Hybribio
